# Identification of 526 Conserved Metazoan Genetic Innovations Exposes a New Role for Cofactor E-like in Neuronal Microtubule Homeostasis

**DOI:** 10.1371/journal.pgen.1003804

**Published:** 2013-10-03

**Authors:** Melissa Y. Frédéric, Victor F. Lundin, Matthew D. Whiteside, Juan G. Cueva, Domena K. Tu, S. Y. Catherine Kang, Hansmeet Singh, David L. Baillie, Harald Hutter, Miriam B. Goodman, Fiona S. L. Brinkman, Michel R. Leroux

**Affiliations:** 1 Department of Molecular Biology and Biochemistry, Simon Fraser University, Burnaby, British Columbia, Canada; 2 Department of Molecular and Cellular Physiology, Stanford University, Stanford, California, United States of America; 3 Department of Cancer Control Research, British Columbia Cancer Research Centre, Vancouver, British Columbia, Canada; 4 Department of Biological Sciences, Simon Fraser University, Burnaby, British Columbia, Canada; University of California San Diego, United States of America

## Abstract

The evolution of metazoans from their choanoflagellate-like unicellular ancestor coincided with the acquisition of novel biological functions to support a multicellular lifestyle, and eventually, the unique cellular and physiological demands of differentiated cell types such as those forming the nervous, muscle and immune systems. In an effort to understand the molecular underpinnings of such metazoan innovations, we carried out a comparative genomics analysis for genes found exclusively in, and widely conserved across, metazoans. Using this approach, we identified a set of 526 core metazoan-specific genes (the ‘metazoanome’), approximately 10% of which are largely uncharacterized, 16% of which are associated with known human disease, and 66% of which are conserved in *Trichoplax adhaerens*, a basal metazoan lacking neurons and other specialized cell types. Global analyses of previously-characterized core metazoan genes suggest a prevalent property, namely that they act as partially redundant modifiers of ancient eukaryotic pathways. Our data also highlights the importance of exaptation of pre-existing genetic tools during metazoan evolution. Expression studies in *C. elegans* revealed that many metazoan-specific genes, including tubulin folding cofactor E-like (TBCEL/*coel-1*), are expressed in neurons. We used *C. elegans* COEL-1 as a representative to experimentally validate the metazoan-specific character of our dataset. We show that *coel-1* disruption results in developmental hypersensitivity to the microtubule drug paclitaxel/taxol, and that overexpression of *coel-1* has broad effects during embryonic development and perturbs specialized microtubules in the touch receptor neurons (TRNs). In addition, *coel-1* influences the migration, neurite outgrowth and mechanosensory function of the TRNs, and functionally interacts with components of the tubulin acetylation/deacetylation pathway. Together, our findings unveil a conserved molecular toolbox fundamental to metazoan biology that contains a number of neuronally expressed and disease-related genes, and reveal a key role for TBCEL/*coel-1* in regulating microtubule function during metazoan development and neuronal differentiation.

## Author Summary

The evolution of multicellular animals (metazoans) from their single-celled ancestor required new molecular tools to create and coordinate the various biological functions involved in a communal, or multicellular, lifestyle. This would eventually include the unique cellular and physiological demands of specialized tissues like the nervous system. To identify and understand the genetic bases of such unique metazoan traits, we used a comparative genomics approach to identify 526 metazoan-specific genes which have been evolutionarily conserved throughout the diversification of the animal kingdom. Interestingly, we found that some of those genes are still completely uncharacterized or poorly studied. We used the metazoan model organism *C. elegans* to examine the expression of some poorly characterized metazoan-specific genes and found that many, including one encoding tubulin folding cofactor E-like (TBCEL; *C. elegans* COEL-1), are expressed in cells of the nervous system. Using COEL-1 as an example to understand the metazoan-specific character of our dataset, our studies reveal a new role for this protein in regulating the stability of the microtubule cytoskeleton during development, and function of the touch receptor neurons. In summary, our findings help define a conserved molecular toolbox important for metazoan biology, and uncover an important role for COEL-1/TBCEL during development and in the nervous system of the metazoan *C. elegans*.

## Introduction

Metazoans, or multicellular animals, represent the epitome of biological complexity. Prerequisite for generating this complexity was the development of a multicellular lifestyle, and the ability to coordinate cell division, migration and differentiation to optimize the overall fitness of the organism [1]. Multicellularity emerged several times during evolution (in algae, plants, fungi and metazoans); however, the one that emerged in metazoans is notable in terms of the extreme diversity of body plans and differentiated cell types that subsequently evolved [2].

**Figure 1:**
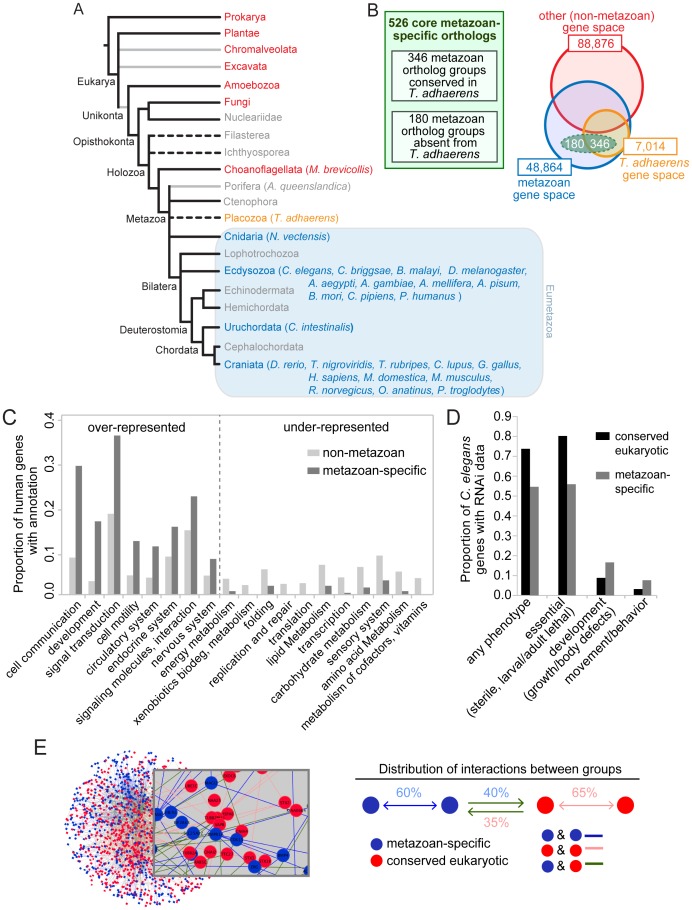
Identification and established functions/interactions of a set of 526 ortholog groups strictly conserved in metazoans. A. Phylogeny of species used to identify the metazoan-specific genes. Grey lines represent possible paraphyletic groups. Dotted lines represent groups of uncertain phylogenetic position. Metazoan groups are colored blue or orange, non-metazoan groups are colored red, and groups that were not included in the analysis are colored grey. All included metazoan species are shown in parentheses; for a complete list of non-metazoan genomes used see Ortho-MCL website. Tree not drawn to scale. B. Comparative genomics approach used to identify the metazoan-specific genes (the ‘metazoanome’). 526 ortholog groups (dashed green oval) were identified on the basis of being conserved in 24 well-sequenced metazoan genomes and absent from 112 well-sequenced non-metazoan genomes. Of these 526 ortholog groups, 346 contained a *T. adhaerens* ortholog (orange circle) and 180 did not. As a comparison to the small number of core metazoan-specific genes, the size of the gene space (total numbers of homologous protein families with at least two members, paralogs included) in metazoan (blue circle) and non-metazoan (red circle) organisms are indicated. Not drawn to scale. C. KEGG functional categories containing disproportionately high or low numbers of human metazoan-specific orthologs. The proportion of metazoan-specific genes belonging to each category is compared with the proportion of human genes in the KEGG database belonging to the same functional category. Categories for which there is a statistically significant difference within a functional category are shown (*p*<0.05 hypergeometric test with Bejamini-Hochburg multiple hypothesis correction). D. RNAi phenotypes of the metazoan-specific genes in *C. elegans*. Fractions of metazoan-specific genes and conserved eukaryotic genes associated with each RNAi phenotype group, are compared. Phenotypic groups are described in the Materials and Methods section. Any phenotype shown relative to the total number of genes with RNAi data. Other phenotypes shown as proportional to the number of genes that display any phenotype. For all four phenotypic groups there is a statistically significant difference between the two sets of genes (Fisher exact test, *p*<0.001). E. Functional interactions analysis. Network representing interactions between metazoan-specific genes, conserved eukaryotic genes and between those two groups. Nodes (representing genes) are colored according to their phylogenetic class: metazoan-specific (blue) and conserved core-eukaryotic (red); edges represent interactions. Distribution of the average proportion of interactions for the two sets of genes. Metazoan-specific and conserved eukaryotic genes interact approximately equally with each other.

Metazoans form a monophyletic group within the opisthokont lineage, a large taxonomic unit containing fungi and several groups of single-celled organisms, including choanoflagellates, ichthyosporea, filasterea and nucleariids [3] (Figure 1A). The properties of the last common ancestor of metazoans and identity of its closest relatives has been the subject of much debate, due to a lack of fossils and sequence data from a broad range of relevant species. This is currently being addressed by genome sequencing efforts such as the UNICORN project [4]. The release of the complete genome sequence of *Monosiga brevicollis*, now widely recognized as the closest known unicellular ancestor of metazoans, as well as the genomes of several early branching metazoan species—including *Amphimedon queenslandica*, *Trichoplax adhaerens* and *Nematostella vectensis*—have provided new insights into the genetic developments underlying metazoan evolution (Figure 1A) [5].

A comparison between the genomes of the sea sponge *A. queenslandica* and the choanoflagellate *M. brevicollis* has highlighted some of the most important genetic innovations that coincided with the metazoan multicellular transition—including those associated with cell growth, proliferation, adhesion, differentiation and immunity [6]. On the other hand, a consistent trend seems to be that exaptation of pre-existing genetic tools played an important role during metazoan evolution. For example, the genome of *M. brevicollis* contains genes associated with a multicellular lifestyle [7], while basal metazoans such as *A. queenslandica* or *T. adhaerens*, which lack differentiated neuronal cells, possess some proteins specifically required for nervous system function in modern eumetazoans (*i.e.* ‘true’ metazoans, excluding Porifera, Ctenophora and Placozoa) [6], [8].

Bearing in mind that limited phylogenetic sampling poses a challenge to classification of early-branching metazoan species, it appears as if basal metazoans lack some of the genetic innovations conserved throughout the eumetazoan lineage. For example, the body plan diversity and number of differentiated cell types within known poriferan species is limited, suggesting that the genetic toolkit possessed by sponges, such as *A. queenslandica*, does not code for the biological complexity displayed by their eumetazoan cousins [6]. Therefore, crucial genetic innovations likely occurred on the eumetazoan stem, after the divergence from a more basal metazoan ancestor, which were essential for the development of differentiated cell types such as neurons and muscle cells, and for overall body plan complexity.

In this study, we sought to uncover genes of central importance to multicellular metazoan biology, and to initiate the analysis of poorly studied or uncharacterized candidates using *C. elegans* as a model system. Employing a comparative genomics approach, we identified 526 metazoan-specific genetic innovations conserved across 24 metazoan species but absent from 112 non-metazoan, mostly single-celled eukaryotic and prokaryotic organisms. These 526 ortholog groups could be considered a set of core metazoan genes which define metazoan biology, since they are both specific to metazoans and highly conserved. As expected, many previously characterized metazoan-specific genes have functions associated with multicellularity. Numerous genes are also characterized by neuronal expression in *C. elegans* and a significant proportion of the human orthologs are linked to human diseases. We highlight 54 core metazoan genes whose biological functions are largely unknown and may represent high-priority targets for understanding fundamental animal biology, and for biomedical research. From our dataset, we chose the poorly studied tubulin folding cofactor E-like (encoded by *TBCEL* in *H. sapiens*, and *coel-1* in *C. elegans*) as a case study for understanding its metazoan-specific character. Our findings reveal a novel role for *C. elegans* COEL-1 during development and in neuronal differentiation and maturation, functions that are unique to metazoans.

## Results

### Identification of core metazoan genes

To uncover the conserved genetic innovations that specifically arose in the metazoan lineage, we identified genes that were highly conserved in metazoans but absent from non-metazoan genomes, including *Monosiga brevicollis*—the closest known unicellular outgroup to metazoans [7] (Figure 1A,B). Our approach is aimed at uncovering the most inclusive set of metazoan ortholog groups while retaining high stringency, and taking into account different levels of genome completeness [9] (Table S1; see also Materials and Methods). Briefly, ortholog predictions for 138 species, including 25 metazoans, were obtained from OrthoMCL-DB [10], [11]. We divided the metazoan species into their phylogenetic clades, and for an ortholog to be classified as metazoan-associated, we required it to be found in nearly all of the well-sequenced species in each metazoan clade, although potentially missing from a few species in separate clades. Also, we ensured that the genes were found exclusively in metazoans, while accommodating a limited number of falsely-predicted non-metazoan orthologs.

Our comparative genomic analysis identified 526 metazoan-specific ortholog groups (Table S2) conserved in a wide array of metazoan species, including *Homo sapiens*, *Drosophila melanogaster* and *Caenorhabditis elegans*. While we refer here to these genes as metazoan-specific, we acknowledge that some may turn out not to be unique to metazoans as more genomes are sequenced. Some species have undergone gene duplication, resulting in multiple proteins per ortholog group. In the 526 ortholog groups, there were 887 human proteins (1.6 proteins/group) compared to only 577 *C. elegans* proteins (1.1 proteins/group) (Table S2). It should be emphasized that while the genes in this dataset are highly conserved in eumetazoans, we did not require them to be conserved in the genomes of basal metazoans such as *A. queenslandica*. Our dataset may therefore contain genetic innovations that evolved in the last common ancestor of eumetazoans, after the divergence of basal metazoans. The recently sequenced placozoan *T. adhaerens* likely represents a phylogenetic intermediate between sponges and cnidaria, and as such, would be the closest known outgroup to the eumetazoans [12]. *T. adhaerens* is a morphologically simple organism with only four described cell types, lacking specialized neurosensory or muscle cells found in eumetazoans [8]. Given the simple morphology and putative position of *T. adhaerens* in the metazoan tree (Figure 1A), we identified ortholog groups that are either present (346) or absent (180) from this genome (Figure 1B, Table S2). The observation that most of our dataset (66%) was robustly conserved in *T. adhaerens* confirms that it is strongly enriched for core metazoan genetic innovations. We further reasoned that differences between these two groups of genes might provide insights into the evolution of biological processes that are unique to eumetazoans.

### Core metazoan genes: Many are uncharacterized and linked to human disease

To provide global insights into the nature of the metazoan-specific genes, we performed several analyses. Namely, we (a) examined whether the genes are characterized to a significant degree or essentially unstudied; (b) positioned annotated human orthologs into functional categories and pathways; (c) examined RNAi phenotypes from published genome-wide *C. elegans* studies; (d) evaluated their involvement within interaction networks; and (e) assessed them for a causative role in human disease.

By searching for existing functional annotations of human genes in the UniprotKB database or *C. elegans* genes in the well-curated WormBase database, we found that 54 of the 526 ortholog groups (∼10%) appear to be completely or largely uncharacterized. This is a smaller proportion than is true for the whole human genome; however, it remains a significant number, considering the highly conserved nature and presumed fundamental biological roles of these genes. We provide this list in Table S3, which includes proteins with a wide range of predicted sequence motifs and domains, and an additional 13 groups that have only been very recently characterized.

As anticipated, human metazoan-specific orthologs with functional annotations were over-represented in functional categories (Figure 1C) and pathways (Table S4) deemed to be important for multicellular animals; these include development, cell-cell communication, and signal transduction. Several organ systems, including nervous, endocrine and circulatory, were also enriched for metazoan-specific genes. In contrast, processes such as DNA replication and repair, transcription, amino acid and carbohydrate metabolism were expectedly under-represented, as most genes implicated in such functions evolved prior to the emergence of the metazoan lineage. In other under-represented categories, such as sensory systems (corresponding in the KEGG database to olfactory, taste and phototransduction), most genes were excluded from our set of core metazoan-specific genes because they are only present in a subset of metazoan species or involve sensory-signaling pathways (e.g., cilium-based) widely conserved across unicellular and multicellular eukaryotes.

When we compared the representation in various categories of metazoan-specific genes with or without a *T. adhaerens* ortholog, glycan biosynthesis and metabolism was the only functional category that showed a significant difference, containing a greater proportion of genes that were absent in *T. adhaerens* (Table S4). This is consistent with respect to previous findings suggesting that proteins associated with the extracellular matrix are in some cases eumetazoan (*i.e.*, Cnidaria and Bilatera) innovations [6]. All other categories, including cell-cell communication, signal transduction, development and nervous system, contained similar proportions of metazoan-specific genes with or without a *T. adhaerens* ortholog (Table S4).

Since *T. adhaerens* lacks a recognizable nervous system, we looked in more detail at the distribution of core metazoan-specific genes in functional pathways associated with the nervous system, namely neuroactive ligand-receptor interactions and axon guidance. A number of metazoan-specific G-protein coupled receptors (GPCRs) involved in human neuroendocrine pathways have orthologs in *T. adhaerens* (Figure S1). These include receptors for glycoproteins (FSHR, LHCGR, TSH), neurotransmitters (GRM1-7, GABBR2) and the neuropeptide galanin (GALR). Similarly, we found that many of the known axon guidance molecules are core metazoan-specific proteins, and several are also found in *T. adhaerens*, including slit, netrin, selected semaphorins, and the receptors robo and eph (Figure S2). However, the axonal guidance machinery is by no means complete in *T. adhaerens*, since several receptors are missing their canonical ligands and vice-versa. These results show that some genes that became associated with modern eumetazoan functions, such as the nervous system, already existed in the last common ancestor of *T. adhaerens* and eumetazoans (see also reference [8]).

We obtained additional insights into metazoan-specific gene function by querying genome-wide *C. elegans* RNAi data. RNAi phenotypes associated with metazoan-specific genes were compared to a control dataset, namely genes with widely conserved eukaryotic orthologs (Figure 1D) (see Materials and Methods for description of the control dataset). Core metazoan genes were collectively less likely to be associated with any particular RNAi phenotype compared with core eukaryotic genes (55% versus 74%). In addition, we classified RNAi phenotypes into three groups: (i) “essential” (embryonic/larval/adult lethality or sterility); (ii) “development” (growth and body shape defects); and (iii) “movement/behavior” (motility and egg-laying defects). Of the genes displaying an RNAi phenotype, the proportion of essential functions was significantly lower for metazoan than for widely conserved eukaryotic genes (56% versus 80%). In contrast, the proportion of genes causing developmental phenotypes and movement/behavior phenotypes was higher for the metazoan-specific group than the conserved-eukaryote group (17% versus 9% and 7% versus 3%, respectively). Therefore, when compared to core eukaryotic genes, core metazoan genes are more likely to cause post-embryonic defects in *C. elegans*. No differences were apparent when comparing metazoan genes with or without a *T. adhaerens* ortholog. Taken together, these results suggest that metazoan-specific genes might have emerged not to perform essential functions, but rather, were used to modify or enhance existing cellular pathways. We note that a potential caveat is that RNAi in *C. elegans* is less effective for genes expressed in neurons, potentially masking important functions of some genes important for this cell type.

To shed light on the functional relationships among core metazoan genes and more ancient eukaryotic genes (using the same control dataset described above), we performed interaction network analyses using the InnateDB database (See Materials and Methods). Our analysis indicates that although metazoan-specific genes are highly connected with each other (60% with one or more connections), they also make extensive connections with ancient eukaryotic genes (40%) (Figure 1E and Figure S3). This supports the idea that the metazoan biological innovations resulted in part from integrating novel components with existing, or evolutionarily more ancient, pathways.

The same evolutionary processes of gene duplication and mutation that drive functional innovation are also to blame for the accumulation of heritable diseases [13]. To estimate what proportion of the human orthologs of metazoan-specific genes are linked to human disease, we queried the curated OMIM (Online Mendelian Inheritance in Man) database. We found that ∼16% (142/887) have a clearly defined link to a documented pathology in humans (Table S5). Diseases linked to the human orthologs of metazoan-specific genes include nervous system disorders (e.g., Parkinson's disease (PARK2/*pdr-1*, PINK1/*pink-1*), Alzheimer's disease (APP/*apl-1*; TAU/*ptl-1*), and torsion dystonia (TOR1A/*tor-1*/*tor-2*/*ooc-5*)), as well as neoplasms, loss of sensory perception, and several other diseases (Table S5). On the whole, these diseases are associated with the proper regulation of cell proliferation and adhesion within certain tissues, and with the development and function of differentiated tissues such as the nervous system. In light of these findings, it is possible that as many as 8 (16% of 54) of the uncharacterized metazoan-specific genes (Table S3) may represent novel biomedical targets.

### Expression analysis of core metazoan genes in *C. elegans*

**Figure 2:**
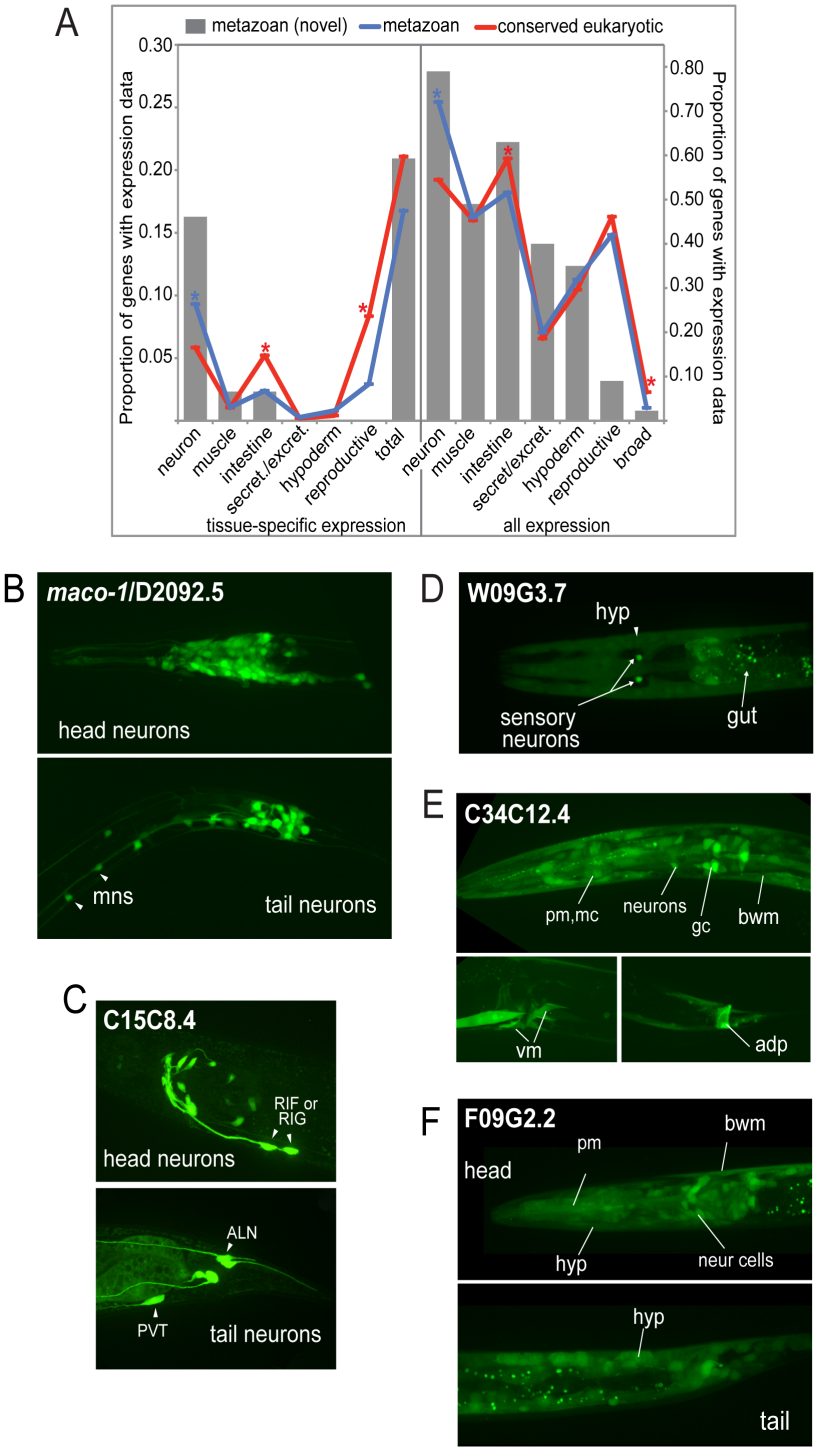
Expression patterns of metazoan-specific genes in *C. elegans*. A. Global analysis of expression patterns of metazoan and widely conserved eukaryotic genes. Expression patterns were categorized as neuronal, muscle, intestinal, secretory/excretory, hypodermal or reproductive, and quantified using GExplore [14]. The left panel shows only tissue-specific expression and the right panel shows all expression. Grey bars show novel metazoan-specific expression patterns determined in this study, blue lines show expression patterns of metazoan-specific genes (previously characterized plus the 43 novel expression patterns collected in this study), and red lines show expression patterns of widely conserved eukaryotic genes. Asterisks indicate statistically-significant differences between metazoan (blue) and conserved eukaryotic (red) genes (*p*<0.05, Fischer's exact test). B–F. Sampling of novel expression patterns of genes investigated in this study. (B) Pan-neuronal expression of D2092.5 *(maco-1)*, ortholog of macoilin, a transmembrane and coiled-coil domain-containing protein. Our data are consistent with studies published during the course of our investigation that showed neuronal-specific expression pattern for this protein and an involvement in neuronal functions in *C. elegans* [42], [43], motoneurons (mns) in the ventral nerve cord are indicated. (C) Neuronal-specific expression of C15C8.4, homolog of LRPAP1 a low-density lipoprotein receptor-related protein tentatively associated with degenerative dementia [77]. Cells expressing C15C8.4 includes RIF or RIG, RIS, 8–10 additional head neurons, and PVT/ALN (a pair of tail neurons), (intestine staining here is unspecific). (D) W09G3.7, homolog of WBSCR16 a predicted RCC1-like nucleotide exchange factor is expressed in a few tissues, hypoderm, intestine and a pair of sensory neurons. (E) C34C12.4, homolog of human C4orf34, is expressed in a subset of neurons in the head, body wall muscle (bwm), intestinal cells, gland cells (gc), vulva muscle and anal depressor (adp). (F) Nearly ubiquitous expression of F09G2.2, a cyclin domain-containing protein homolog to human C2orf34; neuronal and non-neuronal cells in head and tail, pharyngeal muscle (pm), body wall muscle, hypoderm, intestinal cells. Except for D2092.5, the genes are functionally uncharacterized.

Reasoning that the expression patterns of metazoan-specific genes might reveal clues regarding their functions, in particular if restricted to particular tissues, we took advantage of the well-developed tools for examining transgenic expression in *C. elegans*. We generated promoter-GFP-bearing transgenic lines to analyze the expression patterns of 43 core metazoan genes lacking expression data (Table S6); these were prioritized by their relative lack of functional characterization and whether or not the human ortholog was implicated in disease. We also compared the previously determined expression patterns of core metazoan genes to those of core (widely conserved) eukaryotic genes (Figure 2A). For simplicity, expression patterns were categorized into neuronal, muscle, intestinal, secretory/excretory, hypodermal and reproductive tissues, and quantified using GExplore [14] (See Materials and Methods).

There was no significant difference in the overall proportion of genes with tissue-specific expression between core metazoan and core eukaryotic genes (Figure 2A, left panel). Thus, single tissue-specific expression *per se* does not appear to be a distinguishing feature of metazoan genetic innovations. However, when we compared the tissue types individually, the proportion of neuronal-specific expression was slightly higher for metazoan genes than eukaryotic genes (9% versus 6%). Fully 16% of our novel metazoan expression patterns were neuron-specific, suggesting a possible bias in our data set or a lack of detailed analysis in genome-wide studies. Examples of such metazoan-specific genes include D2092.5/macoilin, a transmembrane and coiled-coil domain-containing protein, which displays pan-neuronal expression (Figure 2B) and C15C8.4/LRPAP1, a low-density lipoprotein receptor-related protein that is expressed in a subset of specific neurons (Figure 2C).

When assessing expression by tissue type regardless of specificity, we observed that neurons expressed a greater proportion of core metazoan genes than core eukaryotic genes (72% versus 54%) (Figure 2A, right panel). Neurons also expressed 79% of the 43 additional metazoan genes examined in this study. For example, W09G3.7/WBSCR16, a predicted RCC1-like nucleotide exchange factor, is expressed in a pair of sensory neurons, and in the intestine and hypodermis (Figure 2D). C34C12.4/C4orf34, a completely uncharacterized gene with a predicted transmembrane domain, is nearly ubiquitously expressed (Figure 2E), as is F09G2.2/C2orf24, an uncharacterized gene with a cyclin domain (Figure 2F). In contrast, intestinal cells expressed a lower proportion of metazoan-specific genes than core eukaryotic genes, whereas secretory/excretory, hypodermal and reproductive tissues all had similar proportions of both types of genes expressed (Figure 2A). There was no significant difference when comparing genes present or absent from *Trichoplax* (data not shown). On the whole, our expression data are consistent with the idea that the unique demands of neuronal cell biology were an important *raison-d'être* for some metazoan-specific genes, and may help reveal other cell-specific functions.

### *C. elegans* cofactor E-like (*coel-1*), a core metazoan gene with developmentally-regulated expression in embryos and differentiated neurons

**Figure 3:**
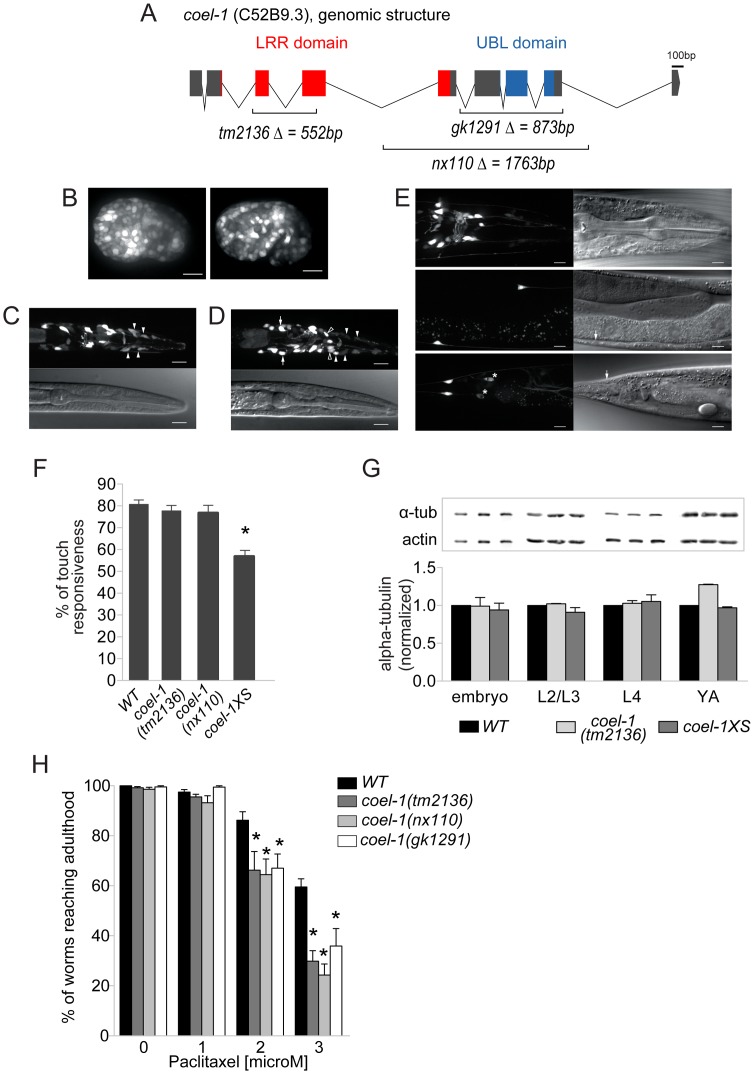
*coel-1* is expressed ubiquitously in embryos but largely confined to neurons in adults, and plays a role in microtubule stability and touch responsiveness. A. Gene structure of *coel-1* (C52B9.3) and nature of the mutant alleles used in this study (*tm2136, gk1291 and nx110*). B–E. Expression of *coel-1* across *C. elegans* development. Transgenic expression of *coel-1* transcriptional GFP reporter in (B) elongating embryos (left panel: early elongation; right panel: comma stage), (C,D) young larvae (white arrows indicate probable expression in the AIZ interneurons, closed arrowheads indicate probable sheath cells, open arrowheads indicate unidentified cells) and (E) head (top panels), body (middle panels) and tail regions (bottom panels) of a mature adult worm. In panel E, note anterior projections from the neurons located laterally along the body wall (ALM) that continue into the head. White arrows indicate cell bodies of probable PLM cells, asterisks show crosstalk into the GFP channel from *sra-6*::dsRED2 in the PVQ neurons. The absence of fluorescence from the nucleus suggests that this signal is in fact derived from the *sra-6*::dsRED2 transgene rather than *coel-1*-driven GFP reporter gene, which contains a nuclear localization signal. In all images, anterior is to the right and ventral is down except for B where anterior is left. Top panels in B and C and right ones in D are GFP images, bottom panels in B and C and left ones in D are DIC images. Scale bar represents 10 µm. F. Worms overexpressing *coel-1 (coel-1XS)* but not *coel-1(tm2136) or coel-1(nx110)* mutant worms have a decreased response to gentle body touch. (n≥30 worms/genotype) *p≤0.001 versus wild-type (WT = N2) (Student's *t*-test). G. Quantitative western blot analyses of total α-tubulin using an antibody directed against all α-tubulins in *C. elegans*. Developmental stages of worms are indicated. Actin represents a loading control. Comparative western blots of *coel-1(tm2136)* mutant worms and *coel-1XS* worms *vs.* wild-type were carried out, and representative blots for each tested stage are shown. Bars are normalized to WT intensity (n≥3). No statistically significant difference is observed (Student's *t*-test p>0.05). H. *coel-1* mutant animals are hypersensitive to taxol, a microtubule-stabilizing anticancer agent. Approximately 100 eggs wild-type (N2), *coel-1(tm2136), (nx110) or gk1291* were incubated at room temperature on plate containing the indicated paclitaxel concentration for 4 days, and worms able to develop to gravid adults were counted. Each genotype was tested in three separate trials; *, *p*≤0.05 Two-way ANOVA followed by Bonferroni post-test. Bars in all panels represent mean ± SEM.

Our expression analysis of metazoan-specific genes uncovered the tubulin folding cofactor E-like gene *TBCEL* as a potential case study for further analysis in *C. elegans*. TBCEL (COEL-1 in *C. elegans*) was first identified based on sequence similarity to tubulin folding cofactor E (TBCE) [15]. The two proteins share UBiquitin-Like (UBL) and Leucine-Rich Repeat (LRR) domains, but TBCEL lacks a cytoskeleton-associated protein-glycine-rich (CAP-Gly) domain present in its counterpart (Figure S4). TBCEL was shown to depolymerize microtubules when overexpressed in cultured cells by committing α-tubulin to proteasomal degradation, while suppression of its activity increased stable microtubule levels [15]. TBCEL is found in all metazoans, including *N. vectensis*, but interestingly, does not appear to be present in *Trichoplax adhaerens*; in contrast, its evolutionary precursor, TBCE, is conserved across all eukaryotes (Figure S4). Given its potential ‘housekeeping’ role in tubulin turnover, we expected the *C. elegans coel-1* gene to be widely expressed across all cell types. Although expressed broadly during embryogenesis (Figure 3B), its expression became restricted to a subset of neurons during larval development (Figure 3C, D) and adulthood (Figure 3E). Co-expression of *coel-1::GFP* with *odr-2::CFP* [16] was observed in the AIZ interneuron (Figure 3D, white arrows), and based on the position of cell bodies relative to those expressing *odr-2::CFP*, other *coel-1*-expressing cells in the head of the animal are likely to be the AVK, AIY, AIM and RIB interneurons, the AWC amphid wing cells, SIBV neurons, OLL neurons and URB neurons. During later stages of larval development, transgenic *coel-1* expression became even more restricted, so that by the adult stage expression was typically observed in ∼10 neurons in the head, the ALM touch receptor neurons along the body wall, and the PLM touch receptor neurons in the tail (Figure 3E). The neuronal-specific expression pattern of *coel-1* was surprising given its presumed general role in microtubule regulation, yet in keeping with a metazoan-exclusive function in *C. elegans*.

### COEL-1 affects microtubule stability

To probe the function of *C. elegans coel-1*, we obtained several mutant strains predicted to interfere with *coel-1* function, including *coel-1(tm2136)* and *coel-1(gk1291)*, and we also generated an additional allele, *coel-1(nx110)*, using Mos insertion mutagenesis (Figure 3A) (see Materials and Methods). The *coel-1*(*tm2136*) allele is predicted to encode a protein with a 77 amino acid deletion in the highly conserved LRR domain (Figure S5). *coel-1(tm2136)* animals do not show any obvious phenotypes; they are viable, fertile, move normally and have a normal life span (data not shown). Both *coel-1(gk1291)* and *coel-1(nx110)* alleles are predicted to remove the C-terminal UBL domain (Figure S5), and also exhibit superficially wild-type development and behavior. Since *coel-1* is expressed in touch receptor neurons (TRNs), we carried out a gentle body touch assay. No significant difference was observed between wild-type and either *coel-1(tm2136)* or *coel-1(nx110)* animals (Figure 3F).

Given previous reports that cofactor E-like could regulate α-tubulin turnover in cultured mammalian cells [15], we examined α-tubulin levels throughout *C. elegans* development. Total, steady-state levels of α-tubulin do not appear significantly different in *coel-1(tm2136)* (Figure 3G) or *coel-1(nx110)* animals (data not shown) relative to wild-type. We then tested our available *coel-1* mutant strains for changes in microtubule function using the microtubule-stabilizing drug paclitaxel (taxol). Eggs hatched on plates containing low doses of paclitaxel arrest their development at larval stages, and the number of animals that escape this arrest to reach adulthood decreases in a dose-dependent manner [17]. All three alleles of *coel-1* (*i.e. tm2136*, *gk1291* and *nx110*) cause a similar degree of hypersensitivity to paclitaxel compared to wild-type animals (Figure 3H). Together, these findings suggest that disruption of *coel-1* function does not affect touch sensitivity or modulate *global* levels of α-tubulin, but that it has an impact on microtubule stability during development.

### Increased COEL-1 activity causes embryonic lethality and influences egg-laying and touch sensitivity

In an attempt to rescue the paclitaxel hypersensitivity of the *coel-1* mutants, and assess the consequence of increased *coel-1* levels, we created an integrated strain carrying additional copies of *coel-1* (*nxIs445*), hereafter referred to as *coel-1XS* (*coel-1* ‘excess’). We confirmed a significant overexpression of *coel-1* from the *coel-1XS* allele at the RNA level by qPCR (Figure S6A). Interestingly, *coel-1XS* animals show a highly variable rate of late-stage embryonic lethality (among individual animals), despite extensive outcrossing. This phenotype has also been observed in animals carrying an extrachromosomal array (*nxEx445*) of the same *coel-1* transgene (Figure S6B). As with *coel-1* mutants, no significant change in the total α-tubulin level was observed in the *coel-1XS* progeny that escape lethality (Figure 3G). However, these animals do have a decreased egg-laying rate (Figure S6C), causing older adults to become full of eggs. We then attempted to test the effect of paclitaxel on *coel-1XS* animals; however, the variable embryonic lethality hindered our ability to carry out the assay. Contrary to *coel-1* mutant animals, *coel-1XS* worms showed a significant reduction in their response to a mechanical stimulus (Figure 3F). Importantly, injection of *coel-1XS* animals with *coel-1* dsRNA (to reduce *coel-1* transcript levels by RNAi) rescued the egg-laying and touch sensitivity defects (Figure S6C, D). Together, these data suggest that the phenotypes observed are due to the overexpression of *coel-1* rather than rearrangement of the transgene upon integration or gene interruption at the integration site. The phenotypic analyses show that overexpression of *coel-1* causes defects in late embryonic development, egg-laying and touch sensation, and that these defects are more severe than *coel-1* disruption. This could be due to the presence of partial redundancy which is able to compensate for lost function, but not able to compensate for vast overexpression. A similar pattern, whereby overexpression is less tolerated than disruption, can be seen with other microtubule regulatory proteins, such as stathmin [18]–[21]. Alternatively, or in addition, the relatively subtle defects caused by the *coel-1* alleles studied here may be due to a partial loss of *coel-1* function.

### COEL-1 disruption or overproduction affect touch receptor neuron development

**Figure 4:**
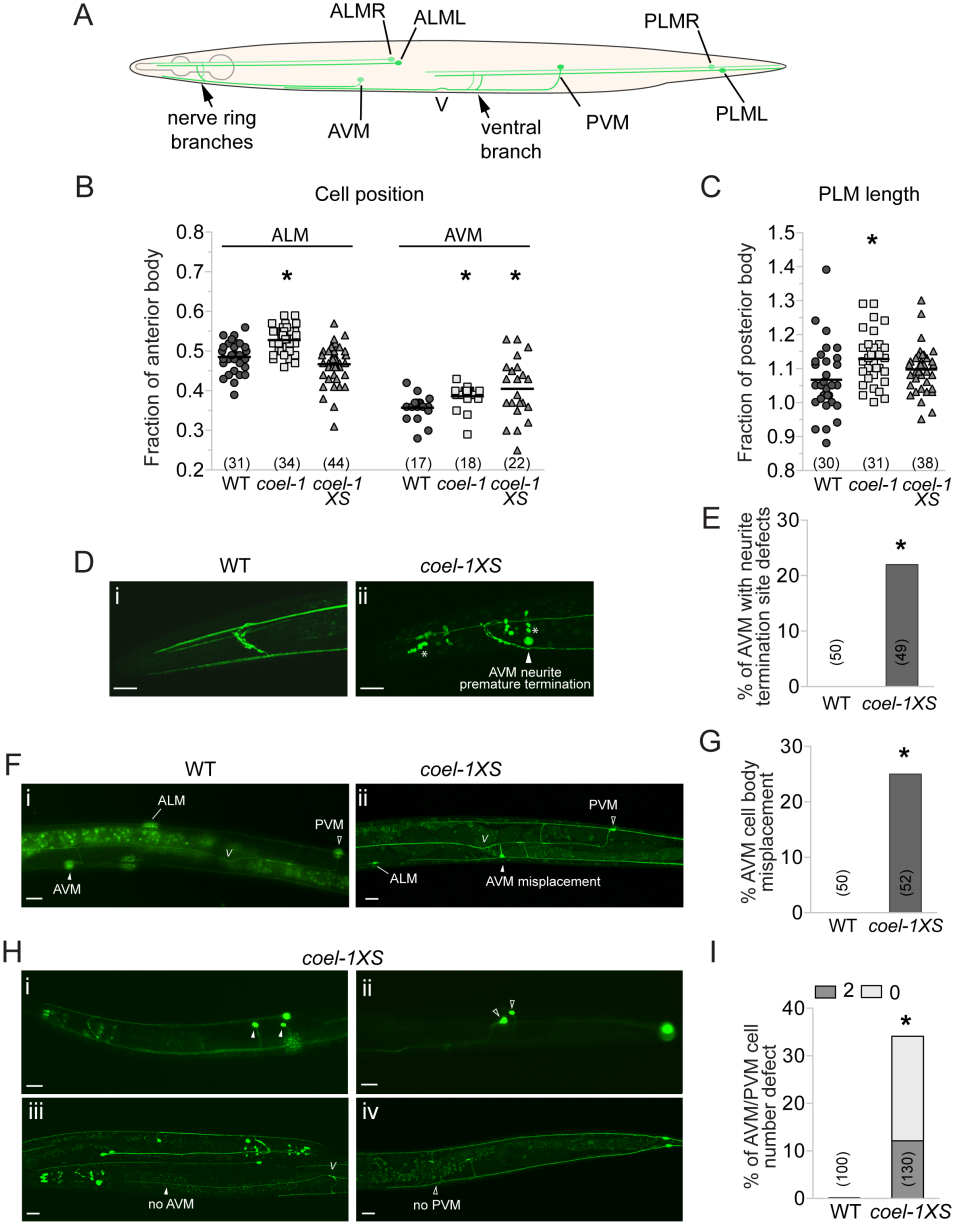
Touch receptor neuron (TRN) morphology, position and number defects in the *coel-1* overexpression and deletion strains. A. Schematic representation of wild-type morphology of the 6 mechanosensory neurons of *C. elegans*. B. ALM and AVM cell bodies are misplaced posteriorly in *coel-1* mutants compared to wild-type animals. Only AVM cell bodies are misplaced in *coel-1XS* animals. Each data point represents the distance from each cell body to the back of the pharynx divided by the length of the anterior body, (i.e., from the tip of the nose to the vulva). C. PLM neurites in *coel-1* mutants are statistically significantly longer than in wild-type. Values indicated represent the length of each PLM neurite divided by the length of the posterior body (i.e., from the vulva to the tail). In panels B and C, the horizontal bar corresponds to the mean of all data points. D. Typical anterior neurite termination site in wild-type worms expressing *zdIs5* (*mec-4*::GFP), a TRN-specific reporter. (i) example of AVM neurite premature termination (arrowhead) at the nerve ring observed in *coel-1XS* animals; (ii) asterisks show crosstalk into the GFP channel from *dpy-30*::dsRED, a co-marker used for the *coel-1* overexpression strain. E. Quantitative analysis of the AVM termination site defect in animals overexpressing *coel-1*. F. In wild-type animals, one AVM and one PVM are observed and AVM is localized anterior to ALM cell bodies. (i) example of AVM mispositioning in *coel-1XS* animals, where AVM is found posterior to the vulva; (ii) Quantification of AVM position defect is presented in G. H. AVM/PVM cell number defect in *coel-1XS* animals. Images shown are of animals with two cells or no cell in AVM (i, iii) and/or PVM (ii, iv); positions are shown and the quantification is reported in I. Scale bar represents 20 µm. V, vulva; WT, wild-type. Brackets indicate the number of neurons measured or scored. *, *p*≤0.05, Fisher exact test.

The response to gentle body touch in *C. elegans* is mediated by the touch receptor neurons (TRNs), and represents a well-studied model system for microtubule-dependent neuronal function [22]. The TRNs consist of two anterior lateral ALM neurons, an anterior ventral AVM neuron, two posterior lateral PLM neurons, and a posterior ventral PVM neuron; each has a single anteriorly-directed process extending from the cell body (Figure 4A). The ALMs are born posterior to the pharynx and migrate to the middle of the animal during embryogenesis, while the AVM and PVM are born post-embryonically and migrate to their final positions during the first larval stage. To examine the role of *coel-1* in the TRNs, where the gene is expressed, we used a *mec-4*::GFP reporter which allowed us to visualize the morphology of these cells in living animals.

Measurements of the total length and cell body positioning of the TRNs revealed several subtle changes in *coel-1 (tm2136)* mutant animals. We observed a significant posterior misplacement of the ALM and the AVM cell bodies in *coel-1* animals compared to wild- type (Figure 4B). We also observed an increased length of the PLM neurons in the *coel-1* animals (Figure 4C), while cell body positioning was normal (data not shown), indicating an overgrowth of their neuronal processes. *coel-1XS* animals also exhibit a significant defect in AVM cell body positioning (Figure 4B,F,G). However, in contrast to the overgrowth of PLM processes in *coel-1* mutant animals, *coel-1XS* animals show a reduced outgrowth of AVM processes, which terminate prematurely (Figure 4D,E). In addition, we observed missing or duplicated neurons for the ventral TRNs in *coel-1XS* animals (Figure 4H,I) and a variety of heterogeneous TRN morphology defects, including disorganized nerve ring branches (data not shown). Similar to other phenotypes detected with the integrated *coel-1XS* transgene, TRN development defects are also observed in worms carrying the extrachromosomal array (Figure S6C). These defects are probably severe enough to result in the partial touch insensitivity that we measured in animals overexpressing *coel-1* (Figure 3F).

AVM and PVM are both descendants of a pair of bilateral Q neuroblasts that each gives rise through asymmetric cell divisions to three different neurons and two apoptotic cells (Figure S6D) [23]. To investigate the possibility that the abnormal ventral TRN cell number observed in *coel-1* overexpressing animals is due to a lineage defect, we looked at AQR and PQR, another pair of neurons arising from the Q neuroblast lineage during post-embryonic development [23]. We found a similar AQR/PQR cell number defect as for AVM/PVM, albeit with an opposite frequency with respect to missing versus extra cells (Figure S6E). The inverse correlation between missing and extra AVM/PVM and AQR/PQR cells suggest a Q neuroblast lineage defect, such that when AQR/PQR are not generated, 2 AVM/PVM are made, and conversely.

Taken together, our results demonstrate that the alteration of the wild-type function of *coel-1* interferes with normal neurodevelopmental processes that control cell fate, cell migration and neurite outgrowth of the TRNs. We did not attempt to address whether the TRN developmental effects of *coel-1* overexpression or disruption were cell autonomous, and it therefore remains strictly possible that the TRN phenotypes we observed are due to *coel-1* function in other cells or tissues. However, this possibility is less likely given the observed TRN expression of transgenic constructs under control of the *coel-1* promoter and because of genetic interactions with genes that are also expressed in the TRNs (i.e., *mec-17* and *atat-2*, see below).

### COEL-1 overexpression reduces microtubule content in the TRNs

To address the potential mechanisms behind the morphological and functional TRN defects we observed, we used serial-section TEM to ask whether *coel-1* function might affect the structure and organization of the microtubule cytoskeleton in these cells. A unique morphological feature of the TRNs is that they contain 15-protofilament microtubules (MTs) arranged in closely packed bundles along their neurites, while most other MTs in *C. elegans* have 11 protofilaments and are not specifically organized [22].

**Figure 5:**
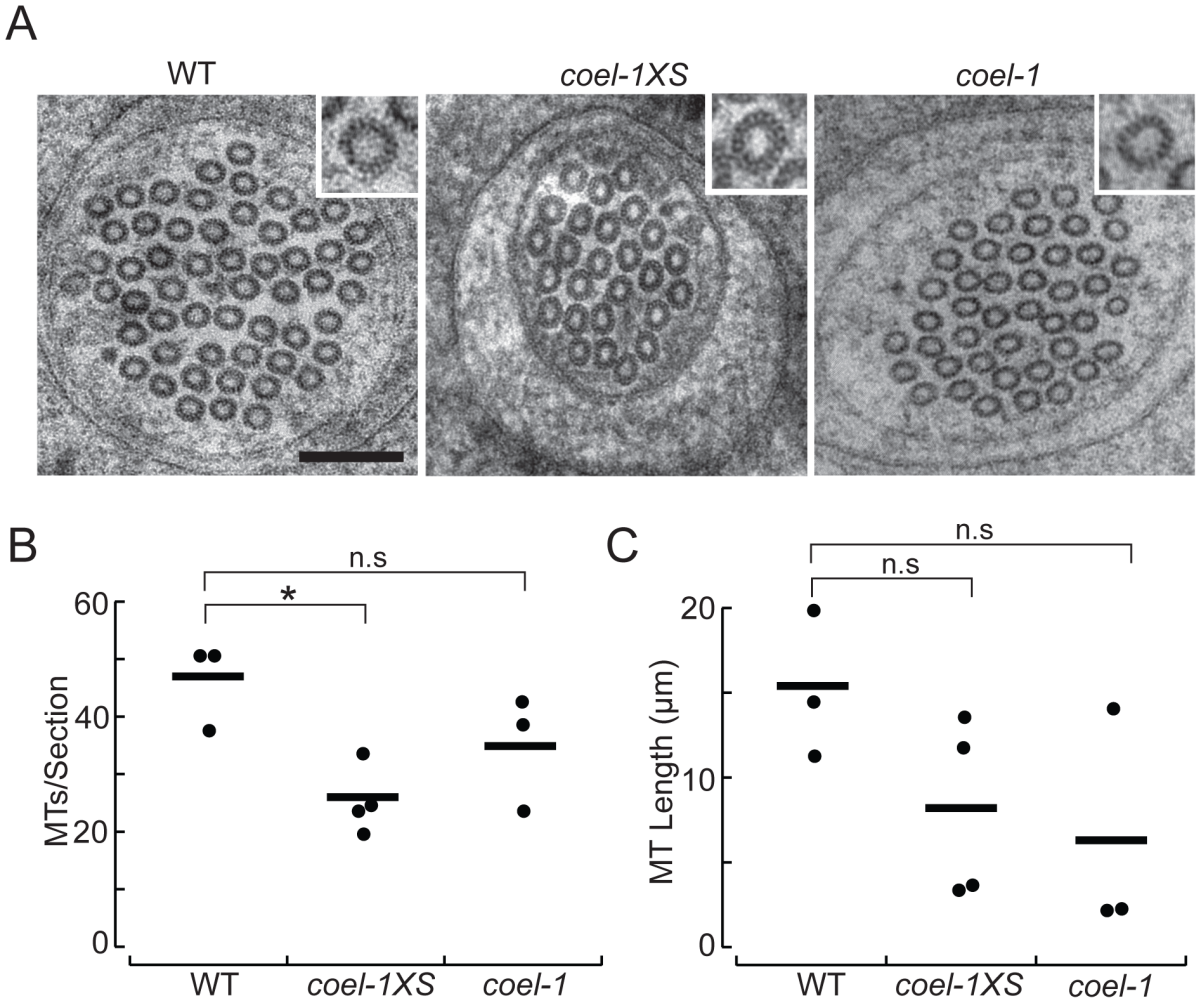
*coel-1* activity influences microtubule number and length, but not protofilament count in PLM touch receptor neurons. A. High-resolution electron micrographs of thin (50 nm) sections of PLM touch receptor neurons in wild-type (WT), *coel-1XS* and *coel-1(tm2136)* animals. Insets show a single microtubule profile, revealing the protofilaments. Scale bar, 100 nm. B. Number of MTs per section as a function of genotype. Bars are the mean values and filled circles are the average values in each dataset. C. Microtubule length as a function of genotype. Bars are the mean and filled circles are the number of serial section datasets tested. Microtubule (MT) length computed from: *L* = 2*Na*/T, where *N* = the average number of MTs/section, *a* = total length of serial reconstruction and *T* = number of MT endpoints observed [25]. In panels B and C, a total of at least 7 µm was reconstructed for each genotype. *, p<0.05, Wilcoxon-Rank test compared to wild-type; n.s, not significantly different.

To visualize individual protofilaments, we prepared wild-type, *coel-1XS*, and *coel-1(tm2136)* mutants using high-pressure freezing and a staining procedure previously developed for this purpose [24]. Using this approach, we found that on average, MTs had the same number of protofilaments (15) in *coel-1*, *coel-1XS* and wild-type PLM neurites (Figure 5A). However, serial reconstructions of neurite segments revealed that *coel-1XS* mutants had significantly fewer microtubules (26±3, mean±s.e.m., *n* = 4 reconstructions, total *L* = 8 µm) than wild-type animals (47±4, mean±s.e.m., *n* = 3, *L* = 7.3 µm) (Figure 5A,B). In contrast, the number of MTs per section in *coel-1(tm2136)* mutants was not statistically different from wild-type (35±6, *n* = 3, *L* = 7.85 µm). In wild-type animals, MTs were 11.4–20.0 µm in length, consistent with previous estimates [24], [25]. In both the *coel-1XS* background and *coel-1* loss of function mutants MTs appeared to be shorter: 3–13.7 µm and 2.3–14.2 µm, respectively. Thus, *coel-1* disruption or *coel-1* overexpression appear to decrease MT length compared to wild-type, but the variance in these datasets was too high to infer a statistically significant effect of genotype on MT length (Figure 5C). These results indicate that *coel-1* overexpression may reduce the MT content in the TRNs, whereas *coel-1* disruption appears to have a more subtle effect on tubulin/MT function. Overall, our TEM data provides a link between cofactor E-like function and neuronal MT homeostasis.

### *coel-1* displays genetic interactions with tubulin acetylation genes

Tubulins are subject to post-translational modifications that participate in fine-tuning the properties of MTs to suit their cellular functions [26]. α-tubulin acetylation at residue K40 is linked to MT stability and function [27]. In *C. elegans*, the only α-tubulin bearing K40 is MEC-12, and acetylated α-tubulin immunoreactivity is found in TRNs, the nerve ring, the VNC and in some ciliated neurons [28]. Given that the phenotypes associated with altered *coel-1* activity are related to MT stability, mechanosensation, neuronal development and MT structure in TRNs, we sought a possible functional link between *coel-1* and tubulin acetylation.

**Figure 6:**
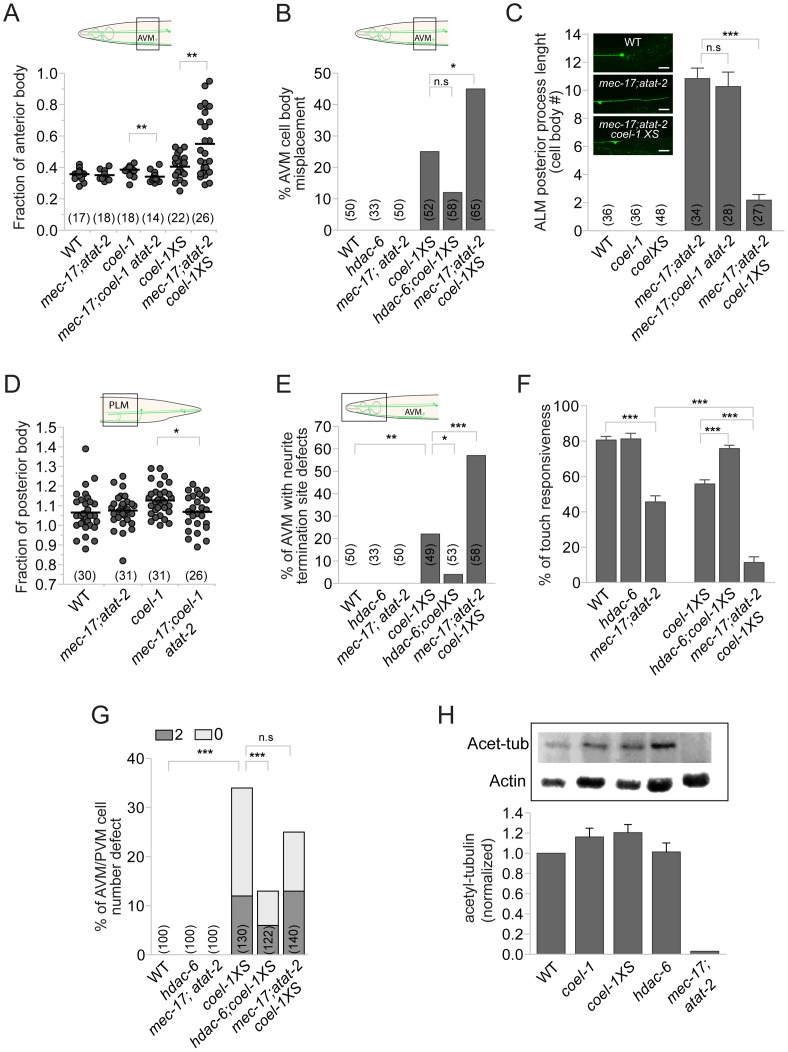
*coel-1* interacts genetically with regulators of tubulin acetylation. A–B. Loss of tubulin acetylation in the *mec-17*;*atat-2* double mutant suppresses the effect of *coel-1* deletion on AVM cell body positioning, and enhances the phenotype caused by overexpression of *coel-1*. C. Images showing that the normally small or non-existent ALM posterior process (top panel) extends abnormally in *mec-17;atat-2* animals (middle panel), a phenotype that is rescued by the overexpression of *coel-1* (bottom panel). The graph shows the quantitation of the average length of the process expressed in cell body equivalent +/− SEM. D. PLM length defect in *coel-1* animals is suppressed when combined with *mec-17;atat-2* mutations. E–F. *hdac-6* and *mec-17;atat-2* mutations respectively suppress, or enhance, the AVM neurite termination defect (E) and the touch sensitivity response (F) observed in *coel-1XS* animals. The mean of touch sensitivity responses ± SEM is represented (n≥30 worms/genotype). G. AVM/PVM cell number defect (extra = 2 or missing = 0) in the *coel-1XS* animals are reduced significantly by the *hdac-6*, but not the *mec-17;atat-2* mutations. H. Quantitative western blot analyses of total acetylated α-tubulin (Acet-tub) using the 6-11B-1 antibody on lysates from young adults. Actin served as loading control and was used to normalize the amounts of protein loaded between samples. Data are normalized to WT (wild-type) intensity (n = 3) and error bars are SEM. No statistically significant difference is observed between *coel-1*, *coel-1XS* or *hdac-6* compared to wild-type. The positive control strain, *mec-17;atat-2*, as expected lacks acetylated tubulin. Brackets indicate the total number of neurons scored. Statistical significances were determined using Student's *t*-test for all panels except B, E and G for which Fisher's exact test was used. *, *p*≤0.05, **; *p*≤0.005; ***, *p*≤0.0005; n.s = not significant.

α-tubulin acetylation is regulated by the balance between acetyltransferases and deacetylases. HDAC6, which is well conserved in *C. elegans*, is a histone deacetylase that can deacetylate α-tubulin K40 [29]. We obtained the *hdac-6(tm3436)* strain, which carries a 476 base-pair deletion spanning exon 4 and intron 4 of *hdac-6* and is superficially wild-type. We found that *hdac-6* animals are very similar to *coel-1* mutant animals. They display subtle TRN morphology defects (posteriorly displaced ALM cell body position and PLM termination sites) (Figure S7A,D) and respond normally to body touch (Figure 6F). The *hdac-6* mutation did not alter the subtle TRN morphology defects associated with the *coel-1(tm2136)* allele (Figure S7A–D). In contrast, the *hdac-6* allele partially rescued most of the phenotypes associated with the *coel-1XS* allele, including TRN morphology defects (Figures 6E,G) and touch insensitivity (Figure 6F). Similarly, the PLM defect associated with the *hdac-6* mutation was reduced by the overexpression of *coel-1* (Figure S7D).

Two *C. elegans* paralogs (*mec-17* and *atat-2*) were recently shown to be redundantly responsible for acetylating the α-tubulin MEC-12 at K40 [30]–[32]. Both single and *mec-17(ok2109)*;*atat-2(ok2415)* double mutant worms have body touch sensitivity defects [30]. As reported by Topalidou and colleagues [33], we found that older *mec-17;atat-2* mutant animals display TRN morphology defects. These subtle AVM and PLM defects, caused by *coel-1* deficiency, were reduced by the *mec-17;atat-2* mutations (Figure 6A,D). In contrast, the acetyltransferase mutations enhanced most of the phenotypes associated with the *coel-1XS* allele, including TRN morphology defects (Figure 6A,B,E) and touch insensitivity (Figure 6F). Notably, we also observed the extension of the normally small or non-existent posterior ALM process that was suppressed by the *coel-1XS* allele, but not the *coel-1(tm2136)* allele (Figure 6C). Altogether, these genetic interactions imply that the activities of *coel-1* and tubulin acetylation regulators overlap in the development and proper function of the touch receptor neurons.

Given the genetic interactions between *coel-1* and regulators of tubulin acetylation, we assessed the relative amounts of acetylated tubulin in different strains by western blot analysis. As previously reported [30], [31], we found that K40-acetylated α-tubulin is detected in protein extracts from wild-type, but not *mec-17;atat-2* mutant worms (Figure 6H). However, no significant change in the levels of acetylated α-tubulin was observed in lysates from *hdac-6*, *coel-1* or *coel-1XS* animals (Figure 6H). Contrary to the complete loss of tubulin acetylation in the *mec-17;atat-2* acetyltransferase-deficient animals, disrupting HDAC-6 appears to have little or no effect on the steady-state level of acetylated tubulin. This could be due, for example, to a partially redundant function with other tubulin deacetylases (e.g., SIRT2; [34]).

In summary, we conclude from our results that *C. elegans* COEL-1 influences microtubule homeostasis in the TRNs, and that its function in these cells relates to at least a subset of acetylated tubulin heterodimers. The requirements for tubulin acetylation/deacetylation and microtubule homeostasis likely vary during different stages of neuronal development and function, helping to account for the diverse effects on cell fate, cell migration, neurite outgrowth and mechanosensory behavior observed upon altering COEL-1 activity.

## Discussion

### Properties of the core metazoan dataset

We have used comparative genomics to identify a core of 526 ortholog groups widely conserved among and unique to metazoans—the ‘metazoanome’. In contrast to previous studies, which have uncovered between 1147 to 1584 animal-specific gene families [5], [6], [35], we did not try to capture all the novelties in the gene repertoire of the metazoan ancestor. Our approach uncovered a much more restricted number of genes that are presumably critical for metazoan biology, by selecting for genes that were innovations in metazoans but also conserved in nearly all well-sequenced metazoan species within the clade. Since we did not require genes to be absolutely conserved in basal metazoans, our dataset includes some genes that may only have emerged in eumetazoans but were then maintained during the evolution of more complex species. As such, our analysis clearly excluded genes that are metazoan-specific but not highly conserved. This is the case, for example, with genes like mdm2, a negative regulator of p53. Mdm2 is conserved from *T. adhaerens* to human but is not found in *C. elegans* or *D. melanogaster* [36], suggesting that elements of the p53 pathway have been lost because they are dispensable in those organisms.

It should be emphasized that, as shown in our results and incorporated into our methodology, incomplete genomes represent a major challenge for the identification of conserved metazoan genes across diverse species. As a greater number of metazoan genome sequences and gene annotation are more robustly completed, additional genes may be identified as meeting the highly conserved metazoan-specific criteria. This is particularly true for the earlier-branching metazoan clades, where very few species have been sequenced. Therefore, our dataset undoubtedly omits some genes that could be of significant interest and significance to metazoan biology. Conversely, as additional genome sequences are obtained of closely related non-metazoans, some genes may need to be removed from this list. However, with the number and breadth of species that can now be investigated, we feel that we have identified a landmark set of genes that can be described as metazoan-specific or metazoan-associated, even though, for the reasons described above, the absolute specificity of the dataset cannot be conclusively determined.

Our global analysis revealed that the core metazoan genes are proportionately less essential than conserved eukaryotic genes (Figure 1D). We also found that, on the whole, core metazoan genes are deployed in multiple differentiated cell types. Our interaction network analysis suggests that metazoan-specific genes interact with each other and with ancient conserved eukaryotic genes (Figure 1E). Taken together, these results are consistent with the notion that a common property of emergent metazoan-specific proteins is that they evolved as partially redundant modifiers of existing cellular processes—in effect modifying these processes in specific cells to create novel functions in a multicellular context. Our work on *C. elegans* cofactor E-like, discussed below, illustrates a protein that emerged at the dawn of eumetazoans to influence pre-existing biological processes, including the microtubule cytoskeleton during embryogenesis, as well as differentiation, migration and neurite outgrowth of a subset of neurons required for behavior (mechanosensation).

### Metazoan evolution: Core metazoan genes and *Trichoplax adhaerens*

Of the core metazoan-specific genes we identified, approximately one-third (34%) were absent from the genome of the basal metazoan *T. adhaerens*, and some of these may therefore have been eumetazoan innovations. However, when we compared those genes with the 346 genes conserved in *T. adhaerens*, we did not find a significant enrichment in any specific functional categories, with the exception of glycan biosynthesis and metabolism, which was underrepresented in *T. adhaerens* (Table S4). Glycans are a diverse group of molecules that are important components of the extra-cellular matrix (ECM), which is essential for many aspects of metazoan biology including cell adhesion, differentiation, morphogenesis and immunity [37]. Heparan sulfate proteoglycan (glypican) GPC6/*gpn-1* is an example of a core metazoan-specific gene that is not found in *T. adhaerens* (Table S2). Although it is possible that the absence of GCP6/*gpn-1* in *T. adhaerens* is due to genome incompleteness or a species-specific gene loss, it is equally plausible that its absence, along with other glycans (*T. adhaerens* does not produce a distinct ECM [38]), reflects an expansion and diversification of genes involved in production and maintenance of the ECM in eumetazoans. Interestingly, the precise roles of glypicans, including GPC6, remain poorly understood. GPC6 has a broad expression pattern which includes the developing brain, and defects in human GPC6 result in omodysplasia (severe limb shortening and facial dysmorphism) [39]. In *C. elegans*, glypican *gpn-1* has been implicated in mediating the proper migration of neuronal precursors [40].

Even in the functional categories associated with higher eumetazoan-specific functions such as the nervous system, the representation of metazoan-specific genes with or without a *T. adhaerens* ortholog did not differ. For example, orthologs of some neuroendocrine G-protein coupled receptors and axon guidance molecules that have evolved specific neuronal functions in eumetazoans can be found in *T. adhaerens*, which otherwise lacks a nervous system (Figure S1, S2). This is consistent with an important contribution of exaptation to the evolution of new metazoan traits [6]. *T. adhaerens* has been shown to exhibit behavioral responses to stimuli [8], and the conserved neuroendocrine pathway components may be part of a primitive stimulus response signaling system that existed in the last common ancestor of *T. adhaerens* and eumetazoans. Furthermore, genetic analyses in *C. elegans* have shown that many of the axon guidance genes have other critical functions (e.g., ephrins and semaphorins in epithelial formation) [41]. This suggests that they could have evolved their axonal guidance activities from a more general cell-cell communication system present in basal metazoans prior to the emergence of the nervous system.

### Uncharacterized core metazoan genes

Of the 526 core metazoan ortholog groups we identified, approximately 10% are uncharacterized or very poorly characterized. For instance, C4orf34/C34C12.4 encodes a small transmembrane domain-containing protein of 99 residues with no functional annotations in any organism, including human, mouse, fly, fish or worm. Its predicted transmembrane domain architecture and our finding that the *C. elegans* ortholog has a broad expression pattern, suggest that it may be involved in a metazoan-specific signal transduction pathway or other conserved cellular process. Importantly, a number of core metazoan genes have been recently characterized (Table S3); these genes are associated with the nervous system, developmental function and human disease. For example, consistent with our expression analysis showing pan-neuronal distribution, macoilin/*maco-1* has been shown to be involved in regulating neuronal functions in *C. elegans* [42], [43]. Another recently characterized metazoan-specific gene is TTC19/*ddl-3*. Its disruption causes mitochondrial complex III deficiency and neurological impairment in humans and flies [44]. These discoveries demonstrate the potential of our dataset as a source of candidate genes for novel functions that may be associated with the nervous system and/or human disease.

In addition, many metazoan-specific proteins, despite having some functional annotation, are poorly characterized and would benefit from analysis from a whole-organism perspective. For example, the protein attractin/mahogany is found in all sequenced metazoans, but its known interaction partner, MC4R, implicated in weight control, has a more restricted distribution [45]. This suggests that attractin/mahogany likely participates in other, potentially broader cellular roles that are yet-to-be-discovered; these could for example be explored in the genetically-amenable metazoan, *C. elegans*, where RNAi of the gene (F33C8.1) suggest roles in fertility and locomotion that may be relevant to synaptic transmission [46]. In this study, we used *C. elegans* to investigate the *in vivo* function of one such poorly characterized metazoan-specific protein, namely tubulin folding cofactor E-like (TBCEL/*coel-1*).

### Cofactor E-like in metazoan development

TBCEL/*coel-1* is evolutionarily related to tubulin folding cofactor E (TBCE), which is conserved across all eukaryotes (Figure S4). In cell culture, TBCEL has been proposed to be a MT-destabilizing factor which disassembles the tubulin heterodimer and promotes the targeting of α-tubulin subunits to the proteasome for degradation [15]. As such, it appears to function together with tubulin folding cofactor E (TBCE) as part of a cellular tubulin quality control machinery that includes other tubulin-specific cofactors, and upstream molecular chaperones (CCT and prefoldin) required for protein folding/assembly [47]. Contrary to the previous cell culture studies on TBCEL, we did not observe a significant change in the overall, or global tubulin levels as an effect of cofactor E-like overexpression in *C. elegans* [15], [48]. This could be due to a more robust function of autoregulatory mechanisms controlling tubulin levels *in vivo* [49]–[51] or the possibility that COEL-1 acts only on a subset of tubulin isotypes. Regardless, we did find that, consistent with previous observations, *C. elegans* COEL-1 does regulate microtubule stability *in vivo*, as indicated by hypersensitivity of *coel-1* mutant animals to the MT-stabilizing drug paclitaxel/taxol (Figure 3H) and the reduced MT number per section in PLM neurites overexpressing *coel-1* (*coel-1XS* strain). These data underscore a net destabilizing role for COEL-1. The drug sensitivity phenotype could arise from a role for COEL-1 in embryonic or early larval development, when *coel-1* is broadly expressed (Figure 3B). A function for COEL-1 in mitotic cells can be also inferred from aberrant AVM/PVM and AQR/PQR cell numbers observed in *coel-1XS* animals (Figure 4J–N), which could potentially be explained by a defect in cell polarity and/or asymmetric cell division [23], [52]. It is possible that additional cell types are missing or duplicated in *coel-1XS* animals. We also found that *coel-1* deficiency altered the final position of ALM and AVM neurons, suggesting a defect in the migration of the ALM neuron during embryogenesis, and the AVM neuron during post-embryonic development. Cell polarity, asymmetric cell division and cell migration are crucial for the development of multicellular animals [53], and the function of *coel-1* in embryos could impact these processes on a broader scale, which could explain the embryonic lethality observed when it is overexpressed (Figure S6B).

### Cofactor E-like in neuronal development and function

During larval and adult development, *coel-1* becomes restricted to neuronal cells, including the TRNs for which we have shown that *coel-1* activity influences cell migration and neurite outgrowth. Neurite outgrowth is the result of growth cone migration and guidance, in a process that is largely analogous to cell migration. These biological processes require the correct balance between microtubule-stabilizing and microtubule-destabilizing forces, as well as efficient microtubule-based transport [54], [55]. In fact, defects in neuronal migration and axon elongation have been associated with disruption of MAP1B and TAU, both microtubule stabilizing proteins [56]. Moreover, microtubule destabilizing factors like members of the stathmin family are also involved in neurite outgrowth and cell migration [20], [57]. Our results indicate that *coel-1* deficiency and *coel-1* overexpression have opposite effects on neurite outgrowth in the TRNs, whereby a subtle overgrowth of the PLM processes in *coel-1(tm2136)* animals was observed; conversely, animals carrying the *coel-1XS* allele displayed premature termination of AVM processes (Figure 4C,E). An opposite effect is also observed for cell migration, whereby the ALM cell body seems to migrate further than their wild-type position when *coel-1* function is deficient, while the AVM cell body migrates less when *coel-1* is overexpressed compared to wild-type. The results are consistent with a microtubule-destabilizing role for *coel-1* necessary for proper cell migration and neurite outgrowth.

The overexpression of *coel-1* decreases microtubule number, supporting a role of COEL-1 as a microtubule destabilizing factor. A plausible mechanism for this would be a tubulin heterodimer binding and disassembly activity for COEL-1, as demonstrated for the human ortholog [15], which, when overexpressed, would push tubulin partitioning from the polymerized microtubule to the free tubulin heterodimer. The functional connections between TRN morphology, their atypical 15-protofilament microtubules and mechanosensory behavior are not yet clear. However, these questions are being addressed [58]. Topalidou et al. [33] have shown for instance that touch response does not depend on the presence of intact 15-protofilament microtubules. In addition, Bounoutas et al. [59] revealed that the polymerization-state of microtubules can regulate protein expression in the TRNs *via* the p38 MAPK pathway. Based on the observation of reduced microtubule content in *coel-1XS* TRN neurites, it is possible that the TRN defects observed upon alteration of this gene are in part, or entirely, a consequence of misregulated expression of factors required for TRN development and function.

There is compelling evidence that the 15-protofilament microtubules of the TRNs are heavily modified by acetylation of α-tubulin at K40. Our data show that both the touch insensitivity and the TRN developmental phenotypes associated with the *coel-1XS* overexpression allele are partially suppressed by mutation of the tubulin deacetylase *hdac-6*, and enhanced by mutations in the acetyltransferases *mec-17* and *atat-2* (Figure 6). Conversely, *hdac-6* has no effect on the TRN developmental phenotypes associated with *coel-1*, while the *mec-17;atat-2* mutations suppress them. In addition, the *coel-1XS* allele, but not the *coel-1(tm2136*) allele, suppressed the *hdac-6* deacetylase PLM phenotype. Collectively, these results suggest that, in the context of the TRN developmental phenotypes, COEL-1 function is antagonistic with respect to tubulin acetylation. However, in light of the recent data showing distinct enzymatic and structural functions for *mec-17* [33] as well as the implications of HDAC-6 in multiple processes in the cell [60], not all the genetic interactions we identified may depend on tubulin acetylation. For example, we found that the *coel-1XS* allele suppressed the posterior ALM process phenotype of *mec-17;atat-2* mutants, a phenotype shown to be independent of *mec-17* enzymatic function.

We propose a general model for COEL-1 function whereby it binds and disassembles tubulin heterodimers, as demonstrated for the human ortholog [15], to recycle and/or degrade specific α-tubulin species (e.g., tubulin isotypes, modified tubulin, damaged tubulin) in cooperation with other factors. Our data suggests that the effects associated with COEL-1 deficiency are relatively minor compared to COEL-1 overabundance. In *coel-1(tm2136*) mutants, a reduction in the turnover or recycling rate of specific tubulin species, for example MEC-12 α-tubulin, could have subtle and diverse effects on microtubule function depending on the cellular and developmental context. In contrast, COEL-1 overabundance in *coel-1XS* animals may exert its more dramatic effect on microtubule stability/polymerization by reducing tubulin heterodimer availability below a critical threshold. The special relationship between COEL-1 function and MEC-12 in the TRNs is supported by our observations that eliminating MEC-12 acetylation (by *mec-17*;*atat-2* mutations) suppressed effects of *coel-1* disruption and enhanced effects of *coel-1* overexpression in these cells. However, given the broad expression of *coel-1* in developing embryos and in a variety of neurons in developing larvae, COEL-1 function may not be specific to MEC-12.

### Metazoan-specific microtubule regulation

Tubulin, and the cellular machinery that regulates its acetylation, were ancient eukaryotic innovations [32], yet the evolution of microtubule regulatory mechanisms is an ongoing process. The microtubule-associated protein 6 (MAP6/STOP) family, for instance, has emerged relatively recently and is only found in vertebrates [61]. In this study, we identified several microtubule-related proteins as highly conserved metazoan innovations, including microtubule-associated protein TAU/*ptl-1*, the echinoderm microtubule-associated proteins (EML1-4/*elp-1*), MIT domain-containing protein 1 (MITD1/Y66D12A.10), microtubule-actin cross-linking factor 1 (MACF1/*vab-10*), and tubulin folding cofactor E-like (TBCEL/*coel-1*). We have shown that *coel-1* acts broadly (i.e., in most cells of the animal) for a brief period during embryonic development and in a subset of differentiated neurons throughout the lifespan of the animal. Notably, both *elp-1* and *ptl-1* are expressed in TRNs of mature *C. elegans* and needed for full touch sensation [62], [63]. *ptl-1* is required for both embryogenesis and touch sensation [62], [63]. The apparently dual roles of these microtubule regulatory factors may simply be indicative of an increased demand for remodeling of the microtubule cytoskeleton in these two situations. Furthermore, the elaboration of microtubule regulatory mechanisms may have been an important part of the evolution of metazoan embryonic development and the emergence of a differentiated neuronal cell type.

In conclusion, this study provides a current best estimate of a core metazoan-specific genetic toolkit (the ‘metazoanome’), as well as an overall assessment of some of its features. The detailed analysis of cofactor E-like in *C. elegans*, revealing a novel role in regulating microtubule homeostasis during development and neuronal differentiation and function, provides an experimentally-validated example of the metazoan-specific character of this dataset. Further study of the uncharacterized or poorly studied core metazoan-specific genes, as well as the interactions between them and with other evolutionarily conserved proteins, should provide important insights into the fundamental biology of multicellular animals and possible targets for neuropathies and other human disorders.

## Materials and Methods

### *C. elegans* strains, molecular biology and genetics

*C. elegans* strains were maintained and cultured at 20°C under standard condition [64]. Constructs were made using standard molecular biology techniques and fusion PCR as previously described [65]. Alleles used in the study include *coel-1(tm2136), coel-1 (nx110), coel-1(gk1291), hdac-6(tm3436)*, *mec-17(ok2109)* and *atat-2(ok2415)*.

RT-PCR analysis of the *coel-1(tm2136)* allele revealed that in contrast to wild-type animals, in which a major transcript of 1300 bp was detected, there were several transcripts of different sizes which were amplified in the *coel-1(tm2136)* mutant worms. Among those transcripts, only one could potentially encode a protein with a deletion of 77 amino acids which would remove the N-terminal half of the LRR domain.

We generated the nx110 allele of *coel-1* using the Mos-mediated mutagenesis [66], we used PCR screen for imprecise excision events from *ttTi16961* worms, which contains a Mos1 transposon inserted on chromosome X. This allele corresponds to a deletion of 1763 bp spanning exons 5 to 8. Before use, this strain was outcrossed at least 4 times. RT-PCR analysis of *coel-1(nx110)* showed that an mRNA is transcribed and is predicted to be translated in a protein lacking the UBL domain in the C-terminal region of COEL-1.

The allele *gk1291* corresponds to an 873 bp deletion including exons 5 to 8 and it is predicted to delete the UBL domain in the C-terminal region of COEL-1.

The following integrated reporter transgenes were used: zdIs5[P*mec-4*::GFP] expressing GFP in the touch receptor neurons, *iaIs19*[P*gcy-32*::GFP] expressing in AQR, PQR and URX neurons and *hdIs26*[*odr-2*::CFP; *sra-6*::dsRED2] expressing fluorescent proteins in neurons (used for cell identification in the *coel-1* expression analysis) [16].

The following transgenes were constructed by PCR and microinjected into worm gonads to generate transgenic lines: (1) *nxEx401*[P*coel-1*::GFP+*dpy-5*(+)], expressing an extrachromosomal transgenic array containing 1621 bp of 5′ sequence containing the putative *coel-1* promoter fused to GFP; several lines were obtained from this injection and showed the same pattern of expression; (2) *nxEx445*[*coel-1(+)+dpy-30*::dsRED] expressing an extrachromosomal transgenic array containing the entire genomic sequence of *coel-1*, including 1621 bp of 5′ sequence and 778 bp of 3′ sequence; three lines were established and one was used for integration by X-ray integration; (3) *nxIs445*, the integrated version of [*coel-1*(+)+*dpy-30*::dsRED], is presumed to be on the X chromosome as *nxIs445* males did not generate any *nxIs445* male progeny during 6X backcrossing.

To confirm *coel-1* overexpression and for molecular characterization of the *coel-1* mutants, N2, *coel-1XS*, *coel-1*(tm2136) and *coel-1*(nx110) cDNAs were isolated by RT-PCR. Briefly, following suspension of mixed-staged worms in Trizol reagent (Invitrogen) and purification with RNeasy (Qiagen), first-strand cDNAs were generated with 1 µg of RNA using the Superscript First-Strand Synthesis System (Invitrogen) with a random hexamer.

For molecular characterization, PCR amplification was performed using primers annealing to the 5′ and 3′ ends of the *coel-1* coding sequence. PCR fragments were incorporated into the PGEM-T Easy vector (Promega) and sequenced. To confirm the overexpression of *coel-1*, real-time qPCR reactions were set-up using the KAPA SYBR FAST master mix (KAPA Biosystem) following the manufacture's protocol. ΔΔCT values were calculated using *cdc-42* and *ama-1* as housekeeping genes [67].

For RNAi, single stranded RNA was synthesized from PCR product containing flanking T7 and SP6 sites using the RiboMax kit (Promega), annealed and injected at ∼1 mg/ml into both gonad arms of young adult animals.

Standard genetic crosses were used to introduce transgenes into different genetic backgrounds and to make double or triple mutant strains. Single-worm PCR reactions were used to genotype the different mutants. A list of all strains generated and used in this study is available in Table S7.

For generation of GFP-reporter transgenic strains, promoter-containing sequences were fused upstream of the GFP-coding region in the pPD95.67 GFP-coding cassette. The PCR constructs were injected into the syncytial region of the gonad. The final concentrations of the injection mix were 10 ng/µl of the target construct along with 100 ng/µl of the marker construct, pCeh361 (*dpy-5*(+)) [68], injected into the target strain *dpy-5(e907)* (CB907). Transgenic F1s (Dpy-5 rescued) were individually plated. Wild-type F2 lines were selected to establish the transgenic lines. When available, we analyzed a second, independent transgenic line.

### Behavioral and MT drug assays

To examine touch responsiveness, each worm was tested 10 times by alternately touching the anterior and posterior with an eyebrow hair [69]. Wild-type animals respond to anterior touch by moving backwards and to posterior touches by accelerating forward. Any worm failing to move a significant distance was counted as a non-response. 30–60 young adults were tested blind to genotype.

To measure the egg-laying rate, worms were reared at room temperature and staged by alkaline/hypochlorite treatment. Equal numbers of young adult worms of each genotype, including *coel-1XS* worms injected with *coel-1* dsRNA, were placed on separate plates and after 36 hours, transferred to fresh plates and incubated at room temperature. Once the progeny on these plates grew to gravid adults, 60 young adult worms were picked onto 3 separate plates (i.e., 20 worms/plate) for each genotype and the number of eggs laid on each plate was counted after 2 hours at room temperature.

To measure embryonic lethality, one-day adult hermaphrodites were allowed to lay eggs at 20°C and removed from plates after 3 hours. The number of eggs laid were counted and monitored every day until hatching.

Worms were tested for their ability to develop into gravid adults in the presence of the microtubule drug paclitaxel (Sigma, T7191; 10 mM in methanol), essentially as previously described [17] except that worms were grown on solid medium instead of liquid.

### Analysis of GFP expression patterns

Mixed-stage transgenic animals were examined for GFP expression using a Quorum WaveFX spinning disc system. Stacks of confocal images with 0.5–1 µm distance between focal planes were recorded, and image acquisition and analyses were done with the Volocity software package (Improvision). Cells were identified by location and morphology in comparison with reference images from Wormatlas (http://www.wormatlas.org/). Maximum intensity projections of all focal planes were used to generate images for the figures.

### Touch receptor neuron anatomy analysis

Touch receptor neurons of young adult worms were visualized by fluorescence microscopy using the *zdIs5* transgene. Both fluorescence and DIC pictures were taken using a Nikon A1R laser scanning confocal system or Zeiss Axioskop 2 microscope. ALM, AVM and PLM total lengths were measured from the cell body to the end of the process. The distances from the vulva to the end of the PLM and from the back of the pharynx to the end of ALM and AVM were also evaluated to examine the site of termination of the processes. Cell body positioning was investigated by measuring the distance from ALM and AVM cell bodies to the back of the pharynx and from PLM and PVM cell bodies to the vulva. To control for individual variation in animal length, the different distances were expressed as ratios with respect to the length of either the anterior body part (tip of the nose to vulva) for ALM and AVM data or the posterior body part (vulva to tail) for PLM and PVM.

We also characterized AVM cell position defect scoring as defective any AVM found at the level of or further than the ALM cell bodies. A premature termination was counted when the anterior process terminates before or at the level of the anterior bulb of the pharynx.

### Immunoblotting

Synchronized worms grown to the appropriate stage on plates at 20°C were collected, suspended in lysis buffer (100 mM Tris, pH 6.8, 4% SDS, 20% glycerol) and treated by 3 cycles of freezing (liquid nitrogen) and boiling. After centrifugation to pellet the insoluble debris, the protein concentration of the supernatants was determined using the BCA protein assay kit (Pierce). Alpha-tubulin and acetylated α-tubulin level were quantitated by fluorescent Western blot. 15 µg of total protein were suspended in Laemmli buffer, separated in 10% SDS-PAGE and electrotransferred to nitrocellulose membranes. Primary antibodies were used at 1∶1000 for the monoclonal mouse anti α-tubulin antibody (Sigma, #T6199) clone DM1A which recognizes amino acids 426–450 of chicken α-tubulins, residues which are highly conserved in *C. elegans* [70]; 1∶500 monoclonal mouse anti acetylated α-tubulin antibody (Sigma, #T7451); 1∶500 polyclonal rabbit anti actin antibody (Sigma, #A2066). Cy5-conjugated goat anti-mouse (GE Healthcare, #PA45009) and anti-rabbit (GE Healthcare, #PA45011) secondary antibodies were used in 1∶2500. Processed blots following manufacturer's protocols were scanned in a Typhoon Phosphorimager system (Molecular dynamics) and quantitated using ImageQuant software (Molecular Dynamics) or ImageJ software (Rasband, W.S., ImageJ, U. S. National Institutes of Health, Bethesda, Maryland, USA, http://rsb.info.nih.gov/ij/,1997-2009).

### Serial-section electron microscopy

Adult nematodes were prepared for EM as previously described [71]. Briefly, animals were frozen using an EMPACT2 high-pressure freezer system (Leica, Vienna, Austria). A Leica AFS freeze substitution apparatus was used to preserve and embed nematodes in 2% glutaraldehyde plus 1% osmium tetroxide and in Eponate 12/Araldite 502. Serial, ultrathin (50 nm) sections were cut with a diamond knife on a Leica Ultracut S microtome and collected on Formvar-coated copper-slot grids. To enhance contrast, sections were post-stained in 3.5% uranyl acetate (30 sec) and Reynold's lead citrate preparation (3 min). The grids were imaged on a transmission electron microscope (JEOL TEM 1230, Tokyo, Japan) and images acquired with an 11 megapixel bottom-mounted cooled CCD camera (Orius SC1000, Gatan, Pleasonton, CA). Images of consecutive sections were aligned manually and analyzed with Reconstruct [72].

### Identification of metazoan-specific orthologs

Our approach utilized the ortholog prediction tool OrthoMCL [10]. OrthoMCL initially predicts orthologous gene pairs by reciprocal best BLAST hit (RBBH) analysis and then clusters the RBBH pairs into highly connected multi-species ortholog groups. Ortholog predictions for 138 species, including 25 metazoan species were obtained from OrthoMCL-DB version 4 [11]. The metazoan species included 11 Craniate species, 1 Urochordate species, 8 Arthropod species, 3 Nematode species, 1 Cnidarian species and 1 Placozoan species (Figure 1, Table S1).

To obtain a list of metazoan-specific ortholog groups from the OrthoMCL groups, groups were selected using two main criteria. First, groups were required to have sufficient coverage across metazoan species. One of the challenges in identifying conserved orthologs across species is accommodating the varying degrees of genome completeness. To avoid missing valid metazoan-specific orthologs in cases where a potentially conserved gene was omitted due to incomplete genome sequence, we performed a cursory assessment of genome completeness in the metazoan species using a list of widely-conserved, low-copy number eukaryotic genes (Table S1). The presence or absence of these conserved eukaryotic genes provides an approximation of the gene coverage [9]. Coverage was on average 94% in the metazoan genomes used in this study, but was observed as low as 79% in the case of *Ciona intestinalis*. To accommodate this source of error, we adopted flexible criteria for selecting metazoan-specific orthologs. We divided the metazoan species into their phylogenetic clades and for an ortholog to be classified as metazoan-specific, we required for it to be found in the majority of species in every metazoan clade, but could be missing from a few species in separate clades. Groups had to have at minimum, orthologs in: 9 of 11 Craniate species, 6 of 8 Arthropod species, 2 of 3 Nematode species. In addition, since *Nematostella vectensis* and *Ciona intestinalis* were sole representatives of their particular phylogenetic clades, we required genes to be conserved in at least one of these genomes. Finally, ortholog groups had to be conserved in a combined total of at least 20 of 24 metazoan species. These criteria ensured a high degree of conservation throughout the metazoan lineage while permitting a limited number of false negatives (i.e. orthologs missing due to errors by OrthoMCL or incomplete genome information).

Because these modified criteria ensures that the gene is strongly represented in all metazoan clades, it is likely that we are more often selecting for groups where an ortholog is absent due to incomplete genome sequence rather than gene loss. The second criterion ensures that the group's orthologs are found exclusively in metazoan species, while accommodating a limited number of falsely predicted non-metazoan orthologs. OrthoMCL is an effective tool for identifying metazoan-specific orthologs. It does, however, generate small numbers of falsely predicted orthologs (false positives). In the OrthoMCL data, singular non-metazoan genes would occasionally be clustered with a group of metazoan-conserved orthologs. These genes would often only be predicted to be orthologs to one or two other metazoan species genes in the group based on existence of an RBBH relationship. From a phylogenetic perspective, it is more likely that these weakly-related, singleton genes are false positives rather than true orthologs. To prevent these groups from being excluded in our list of metazoan-specific orthologs, a second criterion was added: an ortholog group could have at maximum, orthologs in 2 of 112 non-metazoan species, provided that those orthologs had 3 or less RBBH connections to the metazoan orthologs in the group. These weakly connected, singular non-metazoan orthologs likely represent false predictions by OrthoMCL.

### Functional analyses of human metazoan-specific genes

Pathways were obtained from InnateDB [73] and functional categories from KEGG Brite database [74]. To identify pathways and functional categories that were over- or under-represented with metazoan-specific human genes, a hypergeometric test was used. Multiple hypothesis correction was performed using a Benjamini-Hochburg procedure and results were considered significant if the corrected *p*-value was less than 0.05. The set of genes used for comparison in this analysis comprised all human genes with pathway or category annotations in these databases. Differences in the proportions of human metazoan-specific genes with or without a *Trichoplax* ortholog in each category were tested using the Fisher's exact test.

### Identification of uncharacterized genes

To determine the genes that were completely uncharacterized we combined several approaches. First, using Genealacart, the batch querying application based on Genecards database (www.genecards.org) [75], we searched for human genes that had no functional annotation from the UniprotKB database. Second, using wormart, the wormbase implementation of Biomart (http://caprica.caltech.edu:9002/biomart/martview/), we identified the *C. elegans* genes that had no description. Next, we compared the results between human and *C. elegans* to obtain a list of uncharacterized genes in those two species. Finally, we searched manually for genes that had no associated papers in Pubmed.

### Conserved eukaryotic genes not exclusive to metazoans

In order to assess metazoan-specific genes characteristics, we compared them with a set of highly conserved eukaryotic genes that were identified on OrthoMCL-DB by selecting genes found in *H. sapiens*, *C. elegans*, *D. melanogaster*, *S. cerevisiae*, *A. thaliana* and absent in prokaryotes. The total number of eukaryote-specific ortholog groups is 1004, consisting of 1237 *C. elegans* and 1630 human genes.

### Functional interactions of human metazoan-specific genes

To analyze and quantify the functional interactions of metazoan-specific genes and compare them to conserved eukaryotic genes, we used the InnateDB database (http://www.innatedb.com) [73]. Specifically we used the “data analysis” page to retrieve experimentally-verified molecular interactions for each gene. This database allowed us to obtain interactions only between genes of the same set. We identified interactions of metazoan-specific genes with each other, interactions of conserved eukaryotic genes with each other and compared them with the list of interactions between metazoan-specific and conserved eukaryotic genes. Differences in the proportion of interactions were tested using the Fisher's exact test. Cytoscape [76] was used for the visualization of the interaction network.

### Analysis of RNAi phenotypes associated with metazoan-specific genes in *C. elegans*

Wormart (http://caprica.caltech.edu:9002/biomart/martview/), the wormbase implementation of Biomart, was used to retrieve the RNAi phenotypes associated with the metazoan-specific and conserved eukaryotic genes. Specifically, the WS220 gene dataset was used and the genes filtered by their “gene ID.” For the second dataset, we used the RNAi dataset and the “phenotype” filter.

To identify genes that have RNAi data we then selected the filter “limit to RNAi that have one or more scored phenotype”. To identify genes that are associated with any RNAi phenotype we chose the filter “limit to RNAi that have one or more observed phenotype”. To identify the genes associated to particular phenotypes, the filters “phenotype annotation includes observed phenotype” have been selected and the wanted phenotypes have been entered in the “limit to phenotype ID” filter case. The phenotypes analyzed were Emb = embryonic lethal, Ste = sterile, Stp = sterile progeny, Lvl = larval lethal and Adl = adult lethal corresponding to the “Essential” category; Gro = slow growth, Lva = larval arrest, Dpy = dumpy, Bmd = body morphology defect, Bli = blistered, Slm = slim, Lon = long, Sma = small, Pvl = protruding vulva, Muv = multivulval for the “Development” category and Unc = uncoordinated, Prl = paralysed, Rol = roller and Egl = egg-laying defective associated with the “Movement/behaviour” category.

### Identification of disease-associated genes

To identify the numbers of disease-associated genes among the metazoan-specific ones, we queried the OMIM database through BioMart and filtered the output to include only diseases whose molecular basis was known (i.e. containing #3 in the Phenotype map key column of the MIM Gene Map output), as described [13].

### Quantitative analysis of transgenic expression in *C. elegans*

The GExplore tool (http://genome.sfu.ca/gexplore/) [14] was used to compare the annotated expression patterns of metazoan-specific and conserved eukaryotic genes in Wormbase. The total number of genes with annotated expression patterns were identified by an ‘expr’ full-text search. For simplicity, anatomical expression patterns were classified as neuronal (nerv OR neuron), muscle (muscle OR myo), intestinal (gut OR intestin), secretory/excretory (gland OR secretory OR excretory), hypodermal (hypoderm OR epiderm) and reproductive (uter OR gonad OR germ OR sperm OR oocyt OR reproduct). This classification scheme necessarily omits certain cell types (e.g., coelemocytes, male-specific sexual organs, etc.) and some overlap exists between categories (e.g., uterine muscle). Nevertheless, due to the limitations of a text search and the variable annotation of expression data, this scheme was deemed sufficient for a crude, global quantitative comparison between sets of genes. Specific patterns of expression (e.g., tissue-specific, combinations of tissues) was determined by simple Boolean searches. Results were plotted as proportions of the number of genes with annotated expression data for each group (i.e., 376 (333 in Wormbase, 43 novel expression patterns in this study) for metazoan-specific genes and 479 for conserved eukaryotic genes). Differences between metazoan-specific and conserved eukaryotic gene sets were tested using Fisher's exact test.

## Supporting Information

Figure S1 Human neuroendocrine G-protein coupled receptors. Genes that are highly conserved metazoan-specific orthologs are colored blue if they have a *T. adhaerens* ortholog, and yellow if they do not have a *Trichoplax* ortholog.
(PDF)

Figure S2 The human axon guidance pathway. Genes that are metazoan-specific orthologs are colored blue if they have a *T. adhaerens* ortholog, and yellow if they do not have a *T. adhaerens* ortholog.
(PDF)

Figure S3 Cytoscape file for visualization of network of functional interactions. Cytoscape is freely available at http://www.cytoscape.org. Network representing interactions between metazoan-specific genes, conserved eukaryotic genes and between those two groups. Nodes (representing genes) are colored according to their phylogenetic class : metazoan-specific (blue) and conserved core-eukaryotic (red); edges represent interactions.
(ZIP)

Figure S4 Tubulin folding Cofactor E and Cofactor E-Like domain structures and phylogenetic distribution. Schematics depicting the protein structures and evolutionary trees of Cofactor E-like (left) and Cofactor E (right) proteins. TBCE (*C. elegans* K07H8.1) likely represents the ancestral protein, present across all eukaryotes, and TBCEL (*C. elegans* COEL-1/C52B9.3) subsequently emerged in metazoans. CAP-Gly, Cytoskeleton-Associated Protein-Glycine-rich domain; LRR, Leucine-Rich Repeat sequence; UBL, UBiquitin-Like domain.
(PDF)

Figure S5 Cofactor E-like multiple sequence alignment. Multiple sequence alignment of Cofactor E-like sequences from diverse metazoans, showing the different domains of the protein. LRR, Leucine-Rich Repeat sequence; UBL, UBiquitin-Like domain. Aque, *A. queenslandica*; Nvec, *N. vectensis*; Cele, *C. elegans*; Dmel, *D. melanogaster*; Apis, *A. pisum*; Drer, *D. rerio*; Xlae, *X. laevis*; Mdom, *M. domestica*; Hsap, *H. sapiens*; Rnor, *R. norvegicus*; Mmus, *M. musculus*.
(PDF)

Figure S6 *coel-1* overexpression strains and its phenotypes. A. *coel-1* gene expression analysis in *coel-1XS* worms (integrated strain carrying additional copies of *coel-1*) compared to wild-type worms by quantitative PCR. B. Frequency of phenotypes observed (embryonic lethality, defect in number of AVM/PVM neurons) in two different *coel-1* overexpression strains: one integrated (*coel-1XS*), and one with extrachromosomal copies (*nxEx445*). C. Worms overexpressing *coel-1 (coel-1XS)* have an egg-laying defect that is rescued by reducing *coel-1* levels by RNAi. Average number of eggs laid per worm in 2 hours representing the egg-laying activity of 20 worms of each genotype, tested in 3 separate trials. The mean ±SEM are represented. Statistically-significant differences calculated with Student's *t*-test are indicated by *, p≤0.001. D. *coel-1XS* worms have a reduced response to gentle body touch that is rescued by *coel-1* RNAi treatment. n = 30 worms/genotype tested in 3 independent trials. The mean ±SEM are represented. Statistically-significant differences calculated with Student's *t*-test are indicated by *, p≤0.0001. E. Q neuroblast cell lineage. QL (left) and QR (right), born on opposite lateral sides, undergo an identical pattern of cell division and generate three different neurons and two apoptotic cells (X) [23]. F. *coel-1*XS animals have an abnormal number of AQR/PQR neurons. Brackets indicate the total number of neurons scored. Statistical significances were determined using Fisher's exact test *, p≤0.0001.
(PDF)

Figure S7 Quantitation of TRN phenotypes. Graphs depicting cell body position of ALM (A) and AVM (B) neurons, entire length of PLM (C), and PLM termination sites (D) for all strains investigated in this study. Statistically significant differences calculated with Student's *t*-test are indicated by *, p≤0.05.
(PDF)

Table S1 Coverage of core eukaryotic genes across the metazoan species used in the comparative genomic analysis.
(PDF)

Table S2 Metazoan-specific ortholog groups identified in the comparative analysis.
(XLS)

Table S3 Uncharacterized metazoan-specific genes and recently characterized genes [42]–[44], [78]–[89].
(XLS)

Table S4 InnateDB pathways containing significant proportions of human metazoan-specific genes.
(PDF)

Table S5 Metazoan-specific genes associated with human diseases.
(XLS)

Table S6 Novel expression patterns of a subset of metazoan-specific genes.
(XLS)

Table S7 List of strains used in the study.
(PDF)
