# Supplementary material for: Identification of 526 Conserved Metazoan Genetic Innovations Exposes a New Role for Cofactor E-like in Neuronal Microtubule Homeostasis
Source: PLoS Genet. 2013 Oct 3;9(10):e1003804. doi: 10.1371/journal.pgen.1003804 (PMC3789837; doi:10.1371/journal.pgen.1003804)
Supplement: Figure S1 — Human neuroendocrine G-protein coupled receptors. Genes that are highly conserved metazoan-specific orthologs are colored blue if they have a T. adhaerens ortholog, and yellow if they do not have a Trichoplax ortholog. (PDF) [file pgen.1003804.s001.pdf]

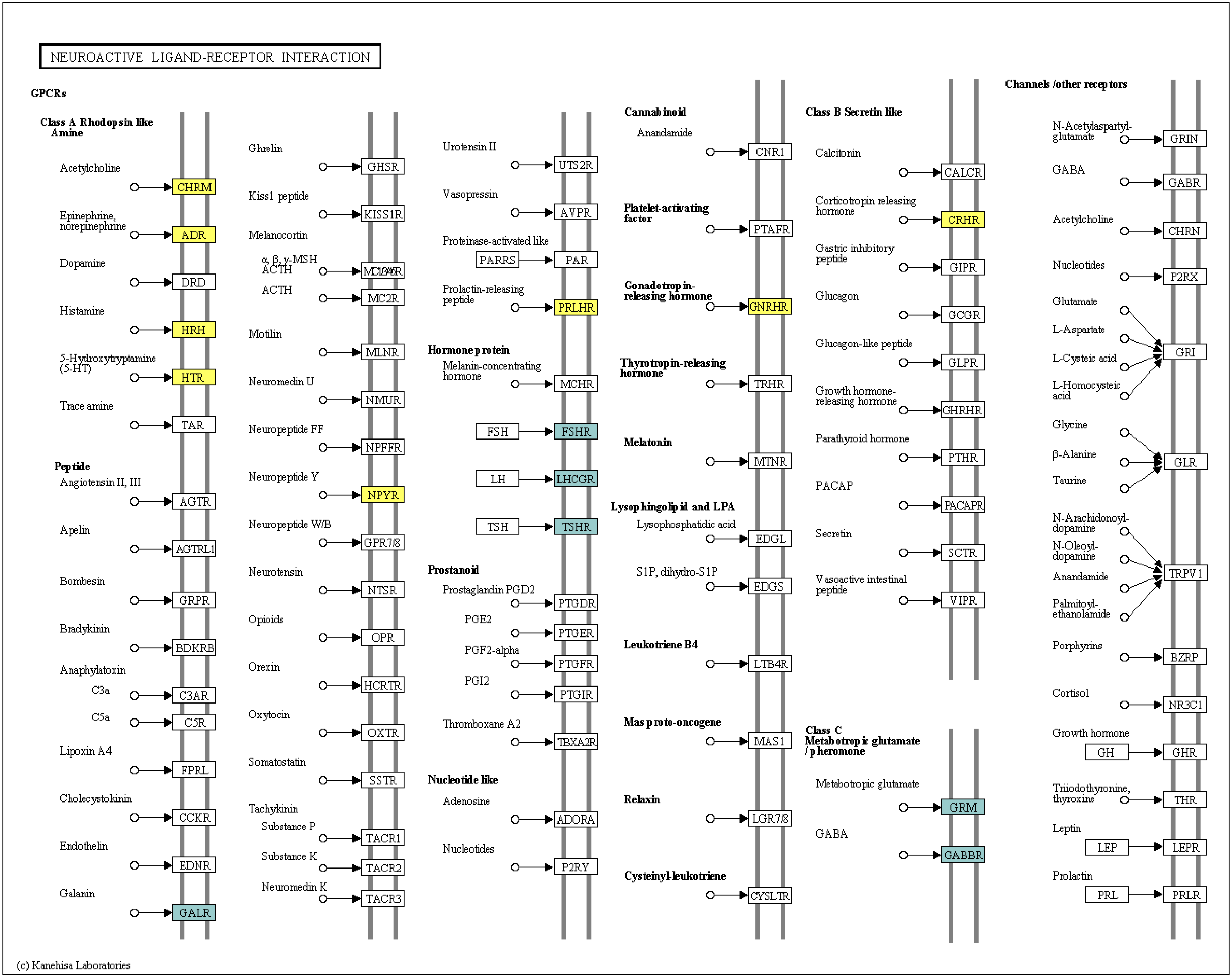

NEUROACTIVE LIGAND-RECEPTOR INTERACTION
GPCRs
Class A Rhodopsin like
Amine
Acetylcholine
CHRM
Epinephrine, norepinephrine
ADR
Dopamine
DRD
Histamine
HRH
5-Hydroxytryptamine (5-HT)
HTR
Trace amine
TAR
Peptide
Angiotensin II, III
AGTR
Apelin
AGTRL1
Bombesin
GRPR
Bradykinin
BDKRB
Anaphylatoxin
C3a
C3AR
C5a
C5R
Lipoxin A4
FPRL
Cholecystokinin
CCKR
Endothelin
EDNR
Galanin
GALR
Ghrelin
GHSR
Kiss1 peptide
KISS1R
Melanocortin
α, β, γ-MSH
ACTH
ACTH
MC2R
Motilin
MLNR
Neuromedin U
NMUR
Neuropeptide FF
NPFFR
Neuropeptide Y
NPYR
Neuropeptide W/B
GPR7/8
Neurotensin
NTSR
Opioids
OPR
Orexin
HCRTR
Oxytocin
OXTR
Somatostatin
SSTR
Tachykinin
Substance P
TACR1
Substance K
TACR2
Neuromedin K
TACR3
Urotensin II
UTS2R
Vasopressin
AVPR
Proteinase-activated like
PARRS
PAR
Prolactin-releasing peptide
PRLHR
Hormone protein
Melanin-concentrating hormone
MCHR
FSH
FSHR
LH
LHCGR
TSH
TSHR
Prostanoid
Prostaglandin PGD2
PTGDR
PGE2
PTGER
PGF2-alpha
PTGFR
PGI2
PTGIR
Thromboxane A2
TBXA2R
Nucleotide like
Adenosine
ADORA
Nucleotides
P2RY
Cannabinoid
Anandamide
CNR1
Platelet-activating factor
PTAFR
Gonadotropin-releasing hormone
GNRHR
Thyrotropin-releasing hormone
TRHR
Melatonin
MTNR
Lysophingolipid and LPA
Lysophosphatidic acid
EDGL
S1P, dihydro-S1P
EDGS
Leukotriene B4
LTB4R
Mas proto-oncogene
MAS1
Relaxin
LGR7/8
Cysteinyl-leukotriene
CYSLTR
Class B Secretin like
Calcitonin
CALCR
Corticotropin releasing hormone
CRHR
Gastric inhibitory peptide
GIPR
Glucagon
GCGR
Glucagon-like peptide
GLPR
Growth hormone-releasing hormone
GHRHR
Parathyroid hormone
PTHR
PACAP
PACAPR
Secretin
SCTR
Vasoactive intestinal peptide
VIPR
Class C Metabotropic glutamate / pheromone
Metabotropic glutamate
GRM
GABA
GABBR
Channels /other receptors
N-Acetylaspartyl-glutamate
GRIN
GABA
GABR
Acetylcholine
CHRN
Nucleotides
P2RX
Glutamate
L-Aspartate
L-Cysteic acid
L-Homocysteic acid
GRI
Glycine
β-Alanine
Taurine
GLR
N-Arachidonoyl-dopamine
N-Oleoyl-dopamine
Anandamide
Palmitoyl-ethanolamide
TRPV1
Porphyrins
BZRP
Cortisol
NR3C1
Growth hormone
GH
GHR
Triiodothyronine, thyroxine
THR
Leptin
LEP
LEPR
Prolactin
PRL
PRLR
(c) Kanehisa Laboratories
