# Supplementary figures and images for: Identification of 526 Conserved Metazoan Genetic Innovations Exposes a New Role for Cofactor E-like in Neuronal Microtubule Homeostasis
Source: PLoS Genet. 2013 Oct 3;9(10):e1003804. doi: 10.1371/journal.pgen.1003804 (PMC3789837; doi:10.1371/journal.pgen.1003804)

# AXON GUIDANCE

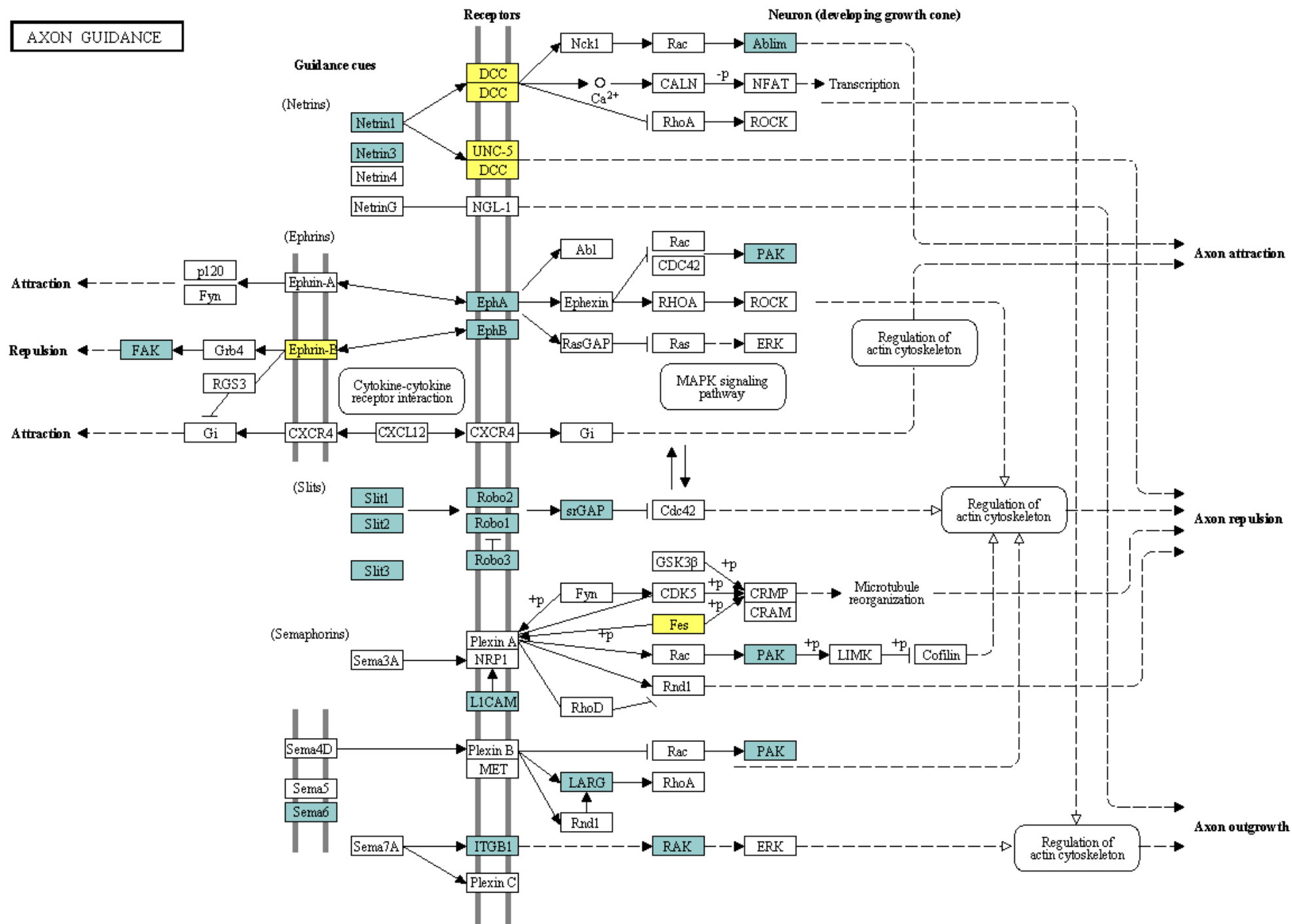

Supplement: Figure S2 — The human axon guidance pathway. Genes that are metazoan-specific orthologs are colored blue if they have a T. adhaerens ortholog, and yellow if they do not have a T. adhaerens ortholog. (PDF) [file pgen.1003804.s002.pdf]

COEL-1(C52B9.3)/TBCEL

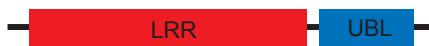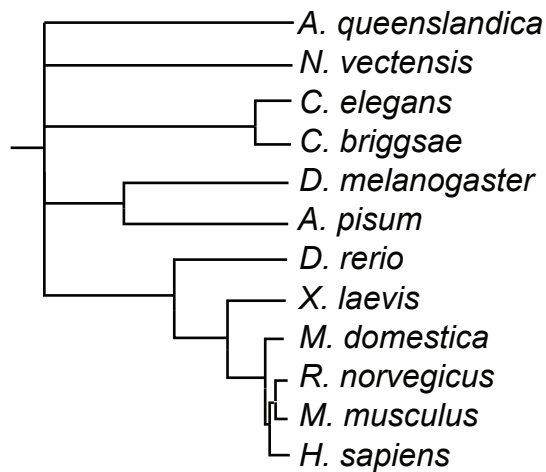

K07H8.1/TBCE

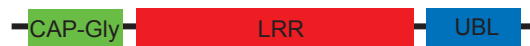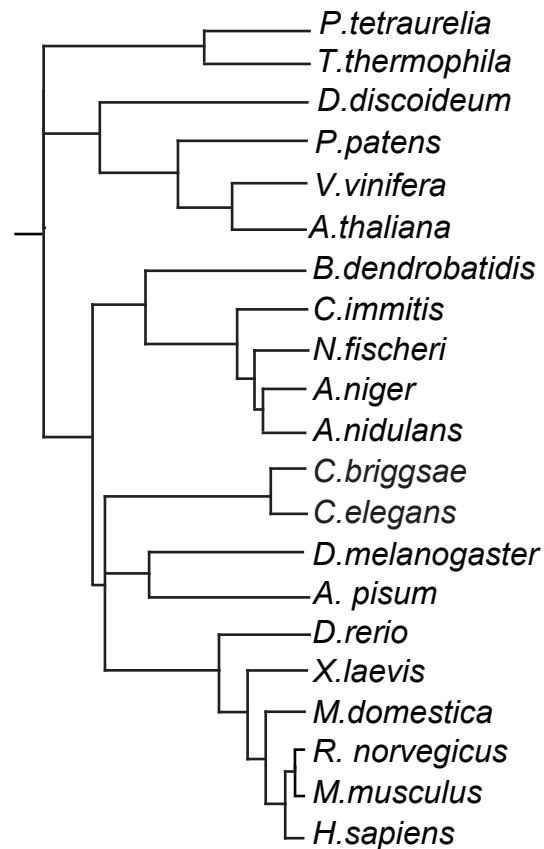

Figure S4

Supplement: Figure S4 — Tubulin folding Cofactor E and Cofactor E-Like domain structures and phylogenetic distribution. Schematics depicting the protein structures and evolutionary trees of Cofactor E-like (left) and Cofactor E (right) proteins. TBCE (C. elegans K07H8.1) likely represents the ancestral protein, present across all eukaryotes, and TBCEL (C. elegans COEL-1/C52B9.3) subsequently emerged in metazoans. CAP-Gly, Cytoskeleton-Associated Protein-Glycine-rich domain; LRR, Leucine-Rich Repeat sequence; UBL, UBiquitin-Like domain. (PDF) [file pgen.1003804.s004.pdf]

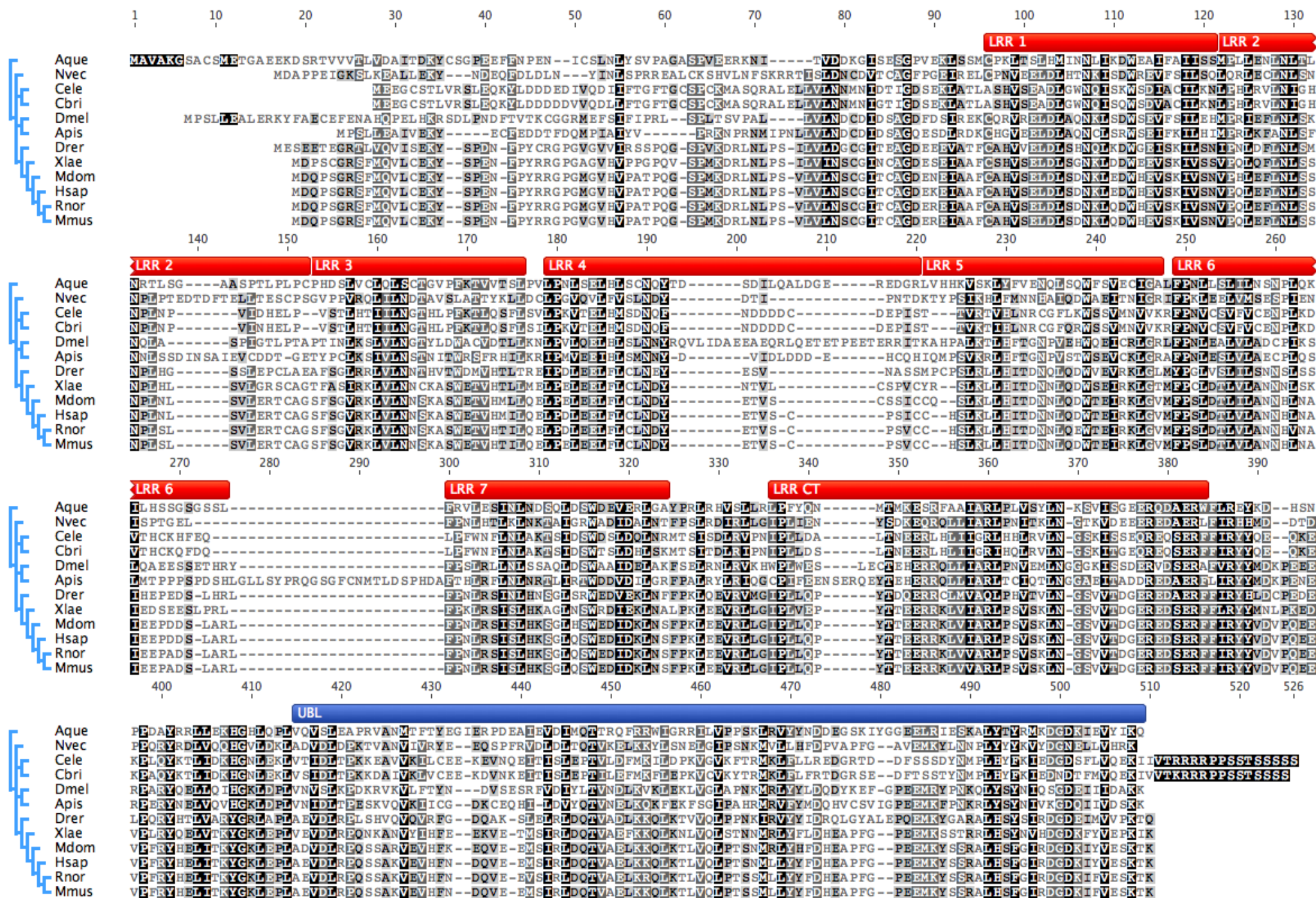

Figure S5

Supplement: Figure S5 — Cofactor E-like multiple sequence alignment. Multiple sequence alignment of Cofactor E-like sequences from diverse metazoans, showing the different domains of the protein. LRR, Leucine-Rich Repeat sequence; UBL, UBiquitin-Like domain. Aque, A. queenslandica; Nvec, N. vectensis; Cele, C. elegans; Dmel, D. melanogaster; Apis, A. pisum; Drer, D. rerio; Xlae, X. laevis; Mdom, M. domestica; Hsap, H. sapiens; Rnor, R. norvegicus; Mmus, M. musculus. (PDF) [file pgen.1003804.s005.pdf]

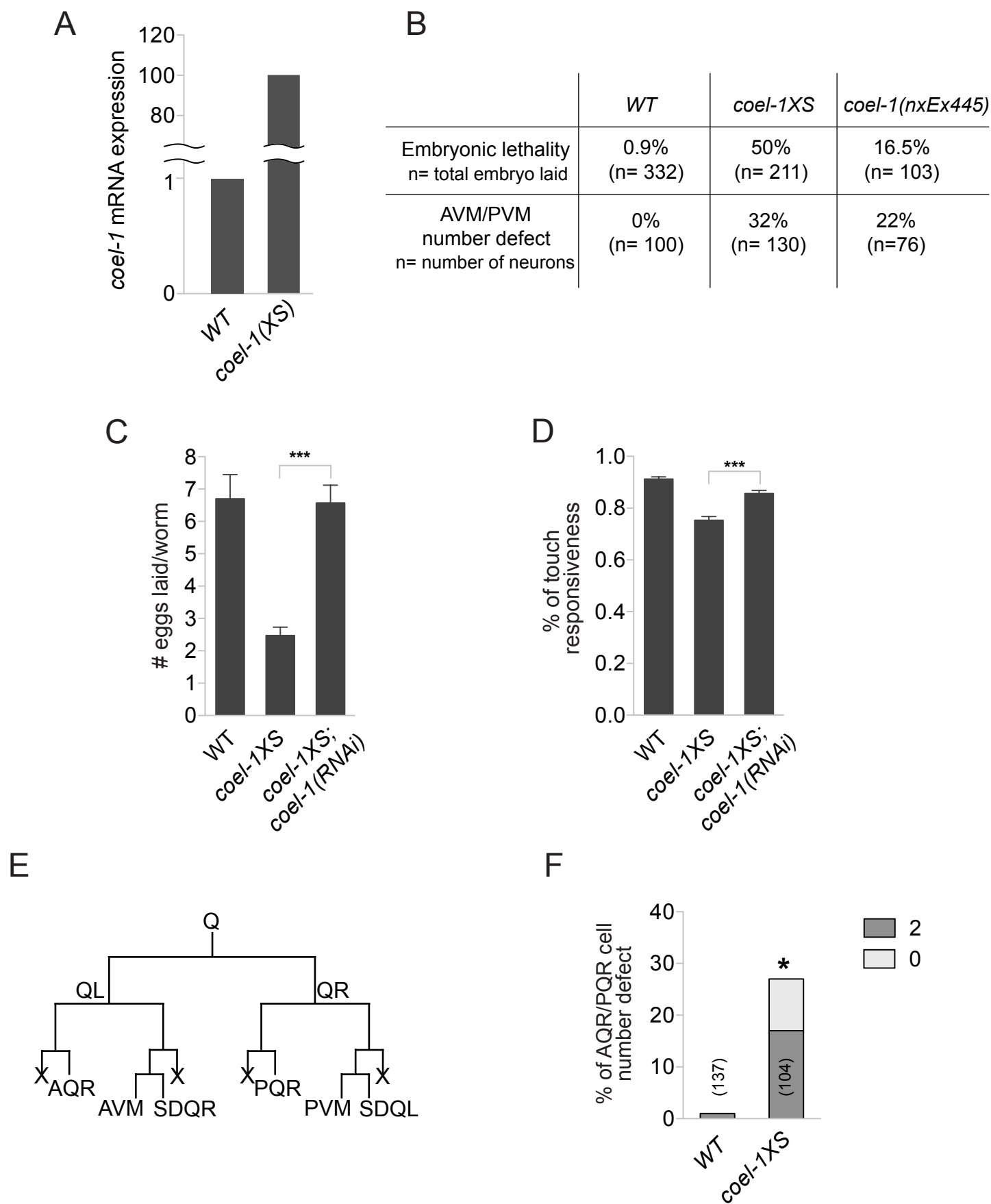

Figure S6

Supplement: Figure S6 — coel-1 overexpression strains and its phenotypes. A. coel-1 gene expression analysis in coel-1XS worms (integrated strain carrying additional copies of coel-1) compared to wild-type worms by quantitative PCR. B. Frequency of phenotypes observed (embryonic lethality, defect in number of AVM/PVM neurons) in two different coel-1 overexpression strains: one integrated (coel-1XS), and one with extrachromosomal copies (nxEx445). C. Worms overexpressing coel-1 (coel-1XS) have an egg-laying defect that is rescued by reducing coel-1 levels by RNAi. Average number of eggs laid per worm in 2 hours representing the egg-laying activity of 20 worms of each genotype, tested in 3 separate trials. The mean ±SEM are represented. Statistically-significant differences calculated with Student's t-test are indicated by *, p≤0.001. D. coel-1XS worms have a reduced response to gentle body touch that is rescued by coel-1 RNAi treatment. n = 30 worms/genotype tested in 3 independent trials. The mean ±SEM are represented. Statistically-significant differences calculated with Student's t-test are indicated by *, p≤0.0001. E. Q neuroblast cell lineage. QL (left) and QR (right), born on opposite lateral sides, undergo an identical pattern of cell division and generate three different neurons and two apoptotic cells (X) [23]. F. coel-1XS animals have an abnormal number of AQR/PQR neurons. Brackets indicate the total number of neurons scored. Statistical significances were determined using Fisher's exact test *, p≤0.0001. (PDF) [file pgen.1003804.s006.pdf]
