# Supplementary material for: Identification of 526 Conserved Metazoan Genetic Innovations Exposes a New Role for Cofactor E-like in Neuronal Microtubule Homeostasis
Source: PLoS Genet. 2013 Oct 3;9(10):e1003804. doi: 10.1371/journal.pgen.1003804 (PMC3789837; doi:10.1371/journal.pgen.1003804)
Supplement: Figure S7 — Quantitation of TRN phenotypes. Graphs depicting cell body position of ALM (A) and AVM (B) neurons, entire length of PLM (C), and PLM termination sites (D) for all strains investigated in this study. Statistically significant differences calculated with Student's t-test are indicated by *, p≤0.05. (PDF) [file pgen.1003804.s007.pdf]

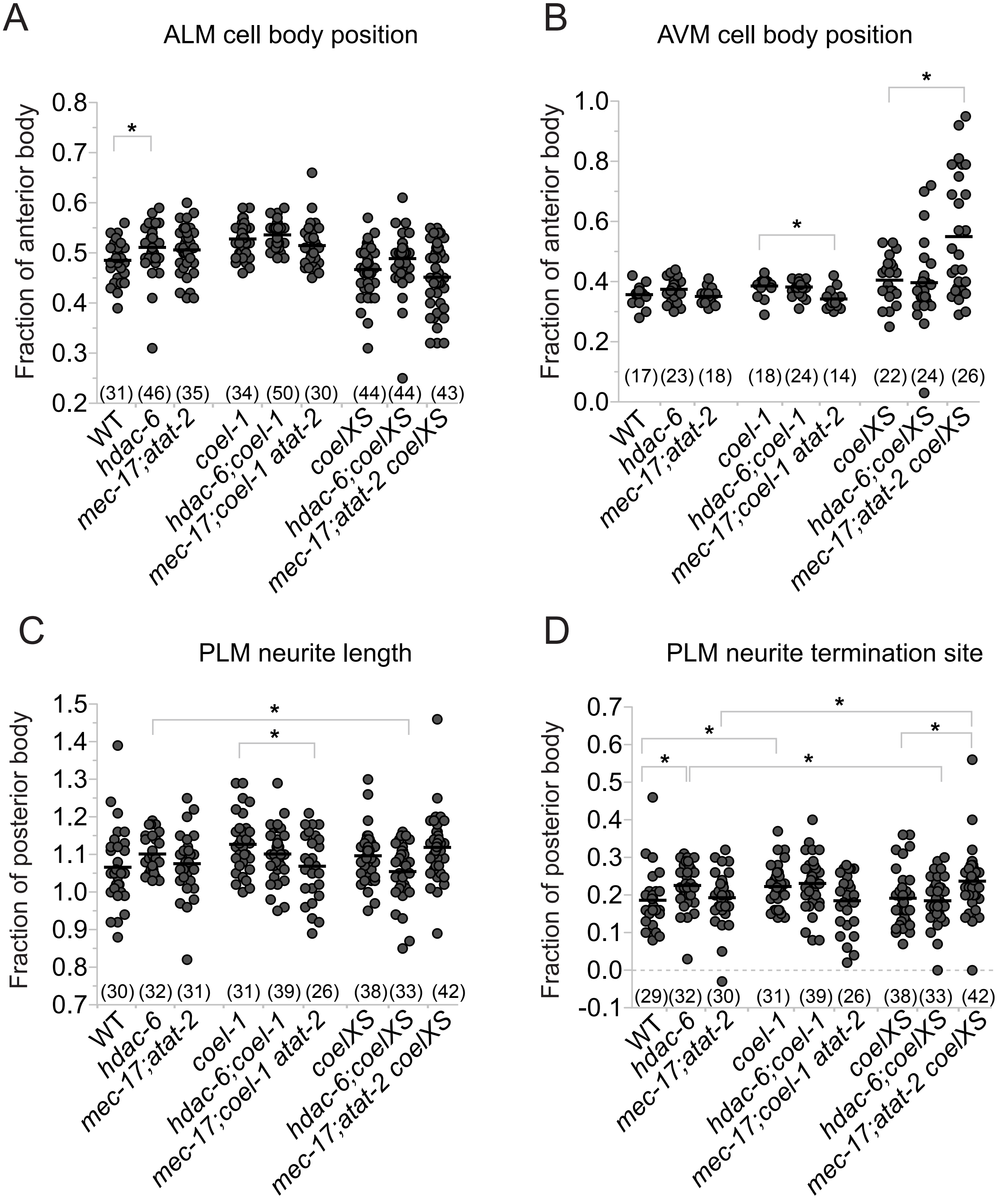
A
ALM cell body position
Fraction of anterior body
0.8
0.7
0.6
0.5
0.4
0.3
0.2
*
(31) (46) (35) (34) (50) (30) (44) (44) (43)
WT
hdac-6
mec-17;atat-2
coel-1
hdac-6;coel-1
mec-17;coel-1 atat-2
coelXS
hdac-6;coelXS
mec-17;atat-2 coelXS
B
AVM cell body position
Fraction of anterior body
1.0
0.8
0.6
0.4
0.2
0.0
*
*
(17) (23) (18) (18) (24) (14) (22) (24) (26)
WT
hdac-6
mec-17;atat-2
coel-1
hdac-6;coel-1
mec-17;coel-1 atat-2
coelXS
hdac-6;coelXS
mec-17;atat-2 coelXS
C
PLM neurite length
Fraction of posterior body
1.5
1.4
1.3
1.2
1.1
1.0
0.9
0.8
0.7
*
*
(30) (32) (31) (31) (39) (26) (38) (33) (42)
WT
hdac-6
mec-17;atat-2
coel-1
hdac-6;coel-1
mec-17;coel-1 atat-2
coelXS
hdac-6;coelXS
mec-17;atat-2 coelXS
D
PLM neurite termination site
Fraction of posterior body
0.7
0.6
0.5
0.4
0.3
0.2
0.1
0.0
-0.1
*
*
*
*
*
(29) (32) (30) (31) (39) (26) (38) (33) (42)
WT
hdac-6
mec-17;atat-2
coel-1
hdac-6;coel-1
mec-17;coel-1 atat-2
coelXS
hdac-6;coelXS
mec-17;atat-2 coelXS

Figure S7
