# Supplementary material for: Identification of 526 Conserved Metazoan Genetic Innovations Exposes a New Role for Cofactor E-like in Neuronal Microtubule Homeostasis
Source: PLoS Genet. 2013 Oct 3;9(10):e1003804. doi: 10.1371/journal.pgen.1003804 (PMC3789837; doi:10.1371/journal.pgen.1003804)
Supplement: Table S1 — Coverage of core eukaryotic genes across the metazoan species used in the comparative genomic analysis. (PDF) [file pgen.1003804.s008.pdf]

1 Metazoan Genome Coverage

Table 1: Percent coverage of conserved eukaryotic genes (CEGs) in the genomes of the metazoan species used in the study. CEGs were isolated following the methodology of Parra *et al.*. Briefly, genes conserved in the well-studied model organisms; *Homo sapiens, Caenorhabditis elegans, Drosophila melanogaster, Saccharyomyces cerevisiae, Arabidopsis thaliana & Schizosaccharomyces pombe* with copy number of one, were considered CEGs. Genomes that were used to isolate the CEGs are highlighted in blue. Genomes whose CEG coverage fall in the lower quartile are highlighted in yellow.

Metazoan Species Genome	Gene Space Coverage (%)
Aedes aegypti	96.1
Anopheles gambiae str. PEST	96.6
Apis mellifera	91.2
Acyrthosiphon pisum	92.4
Brugia malayi	96.1
Bombyx mori	91.7
Caenorhabditis briggsae AF16	98.5
Caenorhabditis elegans	100.0
Ciona intestinalis	79.0
Canis lupus familiaris	98.8
Culex pipiens	94.9
Drosophila melanogaster	100.0
Danio rerio	96.6
Gallus gallus	88.3
Homo sapiens	100.0
Monodelphis domestica	97.6
Mus musculus	100.0
Nematostella vectensis	97.3
Ornithorhynchus anatinus	84.1
Pediculus humanus	97.8
Pan troglodytes	98.3
Rattus norvegicus	96.8
Schistosoma mansoni	91.0
Trichoplax adhaerens	95.9
Tetraodon nigroviridis	95.9
Takifugu rubripes	96.1
