# Supplementary material for: Identification of 526 Conserved Metazoan Genetic Innovations Exposes a New Role for Cofactor E-like in Neuronal Microtubule Homeostasis
Source: PLoS Genet. 2013 Oct 3;9(10):e1003804. doi: 10.1371/journal.pgen.1003804 (PMC3789837; doi:10.1371/journal.pgen.1003804)
Supplement: Table S4 — InnateDB pathways containing significant proportions of human metazoan-specific genes. (PDF) [file pgen.1003804.s011.pdf]

Supplementary Table 4: Human pathways that contain significantly large proportions of metazoan-specific genes (corrected p-value < 0.05). Human metazoan-specific genes are categorized based on having / lacking a *Trichoplax adherens* ortholog. The number of genes that are not conserved across metazoans or are found in non-metazoans are also listed for each pathway. Over-representation statistics were computed using a hypergeometric test with a Benjamini-Hochburg multiple hypothesis correction. Pathways were obtained from InnateDB; a database that has integrated pathways from numerous other sources.

InnateDB Pathway	Original Source DB	Metazoan-specific + *T.adherens* ortholog		Metazoan-specific - *T.adherens* ortholog		Non-conserved / non-metazoan
		# Genes	P-value	# Genes	P-value	# Genes
Cell Communication & Adherence Pathways						
Tight junction	KEGG	23	5.11e-08	4	3.3e-01	103
Focal adhesion	KEGG	23	5.27e-05	12	5.42e-03	163
Adherens junction	KEGG	12	4e-04	5	6.24e-02	57
Apoptotic cleavage of cell adhesion proteins	REACTOME	5	4.48e-04	0	1e+00	6
Nephrin/Neph1 signaling in the kidney podocyte	PID NCI	7	4.53e-04	2	1.86e-01	16
Gap junction	KEGG	13	5.05e-04	3	3.47e-01	73
E-cadherin signaling in the nascent adherens junction	PID NCI	7	4.21e-03	1	5.6e-01	28
Integrin cell surface interactions	REACTOME	8	8.77e-03	7	1.83e-03	39
ECM-receptor interaction	KEGG	10	1.18e-02	7	1.27e-02	67
Arf6 trafficking events	PID NCI	6	2.32e-02	0	1e+00	33
A4b1 and a4b7 Integrin signaling	PID NCI	2	2.57e-02	0	1e+00	1
Tight junction interactions	REACTOME	4	2.65e-02	1	3.94e-01	13
Nectin adhesion pathway	PID NCI	5	2.8e-02	2	2.09e-01	22
N-cadherin signaling events	PID NCI	5	3.5e-02	3	8.2e-02	23
Cell to cell adhesion signaling	PID BIOCARTA	3	4.05e-02	1	3.13e-01	7
A6b1 and a6b4 Integrin signaling	PID NCI	5	1e-01	5	1.35e-02	34
Integrin signaling pathway	PID BIOCARTA	4	1.2e-01	4	2.62e-02	24
Alpha6Beta4Integrin	NETPATH	5	1.78e-01	5	2.86e-02	45
PECAM1 interactions	REACTOME	1	3.74e-01	2	3.73e-02	3
Cell Death Pathways						
Apoptotic cleavage of cellular proteins	REACTOME	4	1.49e-02	2	1.06e-01	9
Fas signaling pathway (cd95)	PID BIOCARTA	4	3.05e-02	0	1e+00	15
Ceramide signaling pathway	PID BIOCARTA	5	4.16e-02	0	1e+00	28

InnateDB Pathway	Original Source DB	Metazoan-specific + *T.adherens* ortholog		Metazoan-specific - *T.adherens* ortholog		Non-conserved / non-metazoan
		# Genes	P-value	# Genes	P-value	# Genes
Breakdown of the nuclear lamina	REACTOME	0	1e+00	2	1.36e-02	1
Cell Motility Pathways						
Regulation of actin cytoskeleton	KEGG	19	5.19e-03	19	1.12e-06	171
Pkc-catalyzed phosphorylation of inhibitory phosphoprotein of myosin phosphatase	PID BIOCARTA	5	8.64e-03	1	4.25e-01	15
Circulatory System Pathways						
Vascular smooth muscle contraction	KEGG	17	8.25e-05	7	4.5e-02	98
Development, Cell Differentiation & Proliferation Pathways						
Signaling by BMP	REACTOME	9	4.6e-07	0	1e+00	10
TGF-beta signaling pathway	KEGG	15	2.27e-05	0	1e+00	71
Wnt	NETPATH	16	5.88e-05	8	1.15e-02	82
BMP receptor signaling	PID NCI	9	4.06e-04	0	1e+00	33
ErbB signaling pathway	KEGG	13	4.29e-04	7	1.36e-02	67
Canonical Wnt signaling pathway	PID NCI	8	7.15e-04	0	1e+00	28
Wnt signaling pathway	KEGG	16	2.72e-03	3	5.78e-01	131
Multi-step regulation of transcription by pitx2	PID BIOCARTA	6	4.81e-03	0	1e+00	21
Alk in cardiac myocytes	PID BIOCARTA	6	4.81e-03	0	1e+00	21
Wnt signaling pathway	PID BIOCARTA	6	7.7e-03	0	1e+00	24
Inhibition of cellular proliferation by gleevec	PID BIOCARTA	5	1.03e-02	1	4.32e-01	16
Glypican 3 network	PID NCI	3	1.77e-02	0	1e+00	5
Presenilin action in Notch and Wnt signaling	PID NCI	6	4.04e-02	0	1e+00	39
EGFR1	NETPATH	14	4.25e-02	6	2.05e-01	155
Notch signaling pathway	KEGG	6	4.47e-02	0	1e+00	41
Signaling events mediated by VEGFR1 and VEGFR2	PID NCI	5	2.17e-01	6	1.23e-02	50
Egf signaling pathway	PID BIOCARTA	2	3.38e-01	3	4.04e-02	15
FGF signaling pathway	PID NCI	3	4.59e-01	6	5.05e-03	39
Vegf hypoxia and angiogenesis	PID BIOCARTA	2	4.88e-01	4	2.18e-02	24
Signaling events mediated by PTP1B	PID NCI	3	4.89e-01	5	2.3e-02	43
PDGFR-beta signaling pathway	PID NCI	3	5.28e-01	6	8.77e-03	46

InnateDB Pathway	Original Source DB	Metazoan-specific + *T.adherens* ortholog		Metazoan-specific - *T.adherens* ortholog		Non-conserved / non-metazoan
		# Genes	P-value	# Genes	P-value	# Genes
Role of erbb2 in signal transduction and oncology	PID BIOCARTA	1	7.48e-01	5	4.12e-03	22
Gab1 signalosome	REACTOME	0	1e+00	3	1.41e-02	8
FGFR1c ligand binding and activation	REACTOME	0	1e+00	2	3.73e-02	4
FGFR3b ligand binding and activation	REACTOME	0	1e+00	2	3.73e-02	4
FGFR3c ligand binding and activation	REACTOME	0	1e+00	2	3.73e-02	4
FGFR4 ligand binding and activation	REACTOME	0	1e+00	2	3.73e-02	4
FGFR1c and Klotho ligand binding and activation	REACTOME	0	1e+00	2	4.74e-02	5
ErbB receptor signaling network	PID NCI	0	1e+00	4	2.39e-03	9
Grb2 events in EGFR signaling	REACTOME	0	1e+00	2	4.74e-02	5
EGFR interacts with phospholipase C-gamma	REACTOME	0	1e+00	2	1.36e-02	1
PDGFR-alpha signaling pathway	PID NCI	0	1e+00	3	4.89e-02	19
Endocrine System Pathways						
GnRH signaling pathway	KEGG	17	6.15e-06	4	2.12e-01	77
Progesterone-mediated oocyte maturation	KEGG	15	1.85e-04	0	1e+00	89
Rapid glucocorticoid signaling	PID NCI	3	1.77e-02	0	1e+00	5
Hormone ligand-binding receptors	REACTOME	3	4.59e-02	1	3.31e-01	8
Glycan Biosynthesis Pathways						
Chondroitin sulfate biosynthesis	KEGG	0	1e+00	4	1.08e-02	18
O-Glycan biosynthesis	KEGG	0	1e+00	4	2.18e-02	26
Immune System Pathways						
Chemokine signaling pathway	KEGG	19	1.68e-03	7	1.51e-01	163
Leukocyte transendothelial migration	KEGG	13	5.02e-03	2	6.47e-01	99
B cell survival pathway	PID BIOCARTA	4	9.64e-03	0	1e+00	9
IL2-mediated signaling events	PID NCI	7	3e-02	1	6.64e-01	47
TNF receptor signaling pathway	PID NCI	6	4.04e-02	0	1e+00	39
BCR	NETPATH	12	4.29e-02	6	1.25e-01	121
Tnfr1 signaling pathway	PID BIOCARTA	3	9.8e-02	3	2.97e-02	11
IL6	NETPATH	3	6.03e-01	6	1.34e-02	57

InnateDB Pathway	Original Source DB	Metazoan-specific + *T.adherens* ortholog		Metazoan-specific - *T.adherens* ortholog		Non-conserved / non-metazoan
		# Genes	P-value	# Genes	P-value	# Genes
T cell receptor signaling pathway	INOH	0	1e+00	4	2.92e-02	30
Nervous System Pathways						
Axon guidance	KEGG	36	7.06e-19	7	5.36e-02	85
Agrin in postsynaptic differentiation	PID BIOCARTA	11	2.93e-05	1	6.2e-01	35
EPHA forward signaling	PID NCI	8	2.79e-04	1	5.2e-01	22
Regulation of Commissural axon pathfinding by Slit and Robo	REACTOME	3	2.44e-03	0	1e+00	1
Ephrin B reverse signaling	PID NCI	6	4.81e-03	3	6.88e-02	18
EPHB forward signaling	PID NCI	7	5.43e-03	2	2.7e-01	29
Long-term potentiation	KEGG	8	2.79e-02	2	4.45e-01	58
Reelin signaling pathway	PID NCI	4	9.39e-02	5	4.46e-03	20
Neuroactive ligand-receptor interaction	KEGG	12	6.36e-01	15	6.32e-03	274
Signal Transduction Pathways						
Adenylate cyclase activating pathway	REACTOME	9	1.3e-09	0	1e+00	2
Adenylate cyclase inhibitory pathway	REACTOME	9	3.6e-09	0	1e+00	3
LPA4-mediated signaling events	PID NCI	9	4.69e-08	0	1e+00	6
G alpha (z) signalling events	REACTOME	8	4.91e-08	0	1e+00	3
PKA activation in glucagon signalling	REACTOME	9	2.82e-07	0	1e+00	9
G alpha (i) signalling events	REACTOME	8	4.74e-07	0	1e+00	6
G(s)-alpha mediated events in glucagon signalling	REACTOME	10	1.19e-06	2	1.99e-01	15
LPA receptor mediated events	PID NCI	14	4.08e-06	6	1.39e-02	44
G alpha (s) signalling events	REACTOME	9	9.12e-06	0	1e+00	17
Endothelins	PID NCI	13	1.04e-05	2	4.08e-01	45
PKA activation	REACTOME	8	1.05e-05	0	1e+00	12
Class C/3 (Metabotropic glutamate/pheromone receptors)	REACTOME	7	2.39e-05	0	1e+00	9
Syndecan-2-mediated signaling events	PID NCI	6	1.18e-02	2	2.35e-01	25
P38MAPK events	REACTOME	3	1.25e-02	0	1e+00	4
Inactivation of Cdc42 and Rac	REACTOME	3	1.77e-02	0	1e+00	5
Regulation of retinoblastoma protein	PID NCI	8	1.88e-02	0	1e+00	55

InnateDB Pathway	Original Source DB	Metazoan-specific + *T.adherens* ortholog		Metazoan-specific - *T.adherens* ortholog		Non-conserved / non-metazoan
		# Genes	P-value	# Genes	P-value	# Genes
MAPK signaling pathway	KEGG	20	2.66e-02	8	2.22e-01	241
Mapkinase signaling pathway	PID BIOCARTA	7	3e-02	0	1e+00	48
P38 signaling mediated by MAPKAP kinases	PID NCI	4	4.14e-02	0	1e+00	17
Ahr signal transduction pathway	PID BIOCARTA	2	4.15e-02	0	1e+00	2
Calcium signaling pathway	KEGG	14	4.25e-02	17	1.51e-06	144
Phospholipase C gamma signaling	INOH	0	1e+00	2	3e-02	3
PLC-gamma1 signalling	REACTOME	0	1e+00	2	3.73e-02	4
