# Supplementary material for: Identification of 526 Conserved Metazoan Genetic Innovations Exposes a New Role for Cofactor E-like in Neuronal Microtubule Homeostasis
Source: PLoS Genet. 2013 Oct 3;9(10):e1003804. doi: 10.1371/journal.pgen.1003804 (PMC3789837; doi:10.1371/journal.pgen.1003804)
Supplement: Table S7 — List of strains used in the study. (PDF) [file pgen.1003804.s014.pdf]

List of strains used in the study

**Strain name**	**Genotype**
MX367	*coel-1(tm2136)X*
MX1094	*coel-1 (nx110)X*
VC3033	*coel-1 (gk1291)X*
MX850	*hdac-6 (tm3436)IV*
GN234	*mec-17(ok2109)IV;atat-2(ok2415)X*
MX496	*N2;nxIs445.C[coel-1(+) + dpy-30::dsRED]X*
MX893	*hdac-6 (tm3436)IV;coel-1(tm2136)X*
MX852	*hdac-6 (tm3436)IV;nxIs445.C X*
MX1457	*mec-17(ok2109)IV;coel-1(tm2136) atat-2(ok2415)X*
MX1458	*mec-17(ok2109)IV;atat-2(ok2415) nxIs445.C X*
MX612	*N2;zdIs5[mec-4::GFP]I*
MX503	*zdIs5 I;coel-1(tm2136)X*
MX639	*zdIs5 I;nxIs445.C X*
MX884	*zdIs5 I;hdac-6(tm3436)IV*
MX894	*zdIs5 I;hdac-6(tm3436)IV;coel-1(tm2136)X*
MX883	*zdIs5 I;hdac-6(tm3436)IV;nxIs445.C X*
MX1428	*zdIs5 I;mec-17(ok2109)IV;atat-2(ok2415)X*
MX1429	*zdIs5 I;mec-17(ok2109)IV;coel-1(tm2136) atat-2(ok2415)X*
MX1430	*zdIs5 I;mec-17(ok2109)IV;atat-2(ok2415) nxIs445.C X*
MX401	*dpy-5(e907);nxEx401[coel-1p::GFP + dpy-5(+)]*
MX635	*nxEx401;hdIs26[odr-2::CFP; sra-6::dsRED2] III*
ZG611	*aIs19[Pgcy-32::GFP + unc-119(+)]*
MX1790	*aIs19 ;nxIs445.C X*
BC11041	dpy-5(e907)/dpy-5(e907);sEx11041(rCes F10C5.2::gfp+PcEH361)
BC11182	dpy-5(e907)/dpy-5(e907);sEx11182(rCes ZK381.5a::gfp+PcEH361)
BC13325	dpy-5(e907)/dpy-5(e907);sEx13325(rCes K02D7.2::gfp+PcEH361)
BC16360	dpy-5(e907)/dpy-5(e907);sEx16360(rCes K05B2.2::gfp+PcEH361)
BC16668	dpy-5(e907)/dpy-5(e907);sEx16668(rCes R01B10.5::gfp+PcEH361)
BC16972	dpy-5(e907)/dpy-5(e907);sEx16972(rCes F45H7.6::gfp+PcEH361)
BC17202	dpy-5(e907)/dpy-5(e907);sEx17202(rCesF40F12.5a::gfp+PcEH361)
BC17327	dpy-5(e907)/dpy-5(e907);sEx17327(rCes B0302.1a::gfp+PcEH361)
BC17957	dpy-5(e907)/dpy-5(e907);sEx17957(rCes R05G6.10::gfp+PcEH361)
BC17958	dpy-5(e907)/dpy-5(e907);sEx17958(rCes C17E4.9::gfp+PcEH361)
BC17960	dpy-5(e907)/dpy-5(e907);sEx17960(rCes C15C8.4::gfp+PcEH361)
BC17961	dpy-5(e907)/dpy-5(e907);sEx17961(rCes C34C12.4::gfp+PcEH361)
BC17964	dpy-5(e907)/dpy-5(e907);sEx17964(rCes C18E9.11::gfp+PcEH361)
BC17965	dpy-5(e907)/dpy-5(e907);sEx17965(rCes F56A8.3::gfp+PcEH361)
BC17967	dpy-5(e907)/dpy-5(e907);sEx17967(rCes K07F5.6::gfp+PcEH361)
BC17969	dpy-5(e907)/dpy5(e907);sEx17969(rCesY62E10A.9::gfp+PcEH361)
BC17973	dpy-5(e907)/dpy-5(e907);sEx17973(rCes F08B12.1::gfp+PcEH361)
BC17975	dpy-5(e907)/dpy-5(e907);sEx17975(rCes F22D6.1::gfp+PcEH361)
BC17978	dpy-5(e907)/dpy-5(e907);sEx17978(rCes C41D11.3::gfp+PcEH361)

!

BC17979	dpy-5(e907)/dpy-5(e907);sEx17979(rCes C33A11.2::gfp+PcEH361)
BC17983	dpy-5(e907)/dpy-5(e907);sEx17983(rCes R17.3::gfp+PcEH361)
BC17990	dpy-5(e907)/dpy5(e907);sEx17990(rCesY37A1B.12::gfp+PcEH361)
BC17992	dpy-5(e907)/dpy-5(e907);sEx17992(rCes C01B7.4::gfp+PcEH361)
BC17998	dpy-5(e907)/dpy-5(e907);sEx17998(rCes D2013.1::gfp+PcEH361)
BC18000	dpy-5(e907)/dpy-5(e907);sEx18000(rCes D2092.5::gfp+PcEH361)
BC18006	dpy-5(e907)/dpy-5(e907);sEx18006(rCes C44C8.6::gfp+PcEH361)
BC18008	dpy-5(e907)/dpy-5(e907);sEx18008(rCes F09G2.2::gfp+PcEH361)
BC18009	dpy-5(e907)/dpy-5(e907);sEx18009(rCes C06A6.3::gfp+PcEH361)
BC18016	dpy5(e907)/dpy5(e907);sEx18016(rCesY46E12BL.4::gfp+PcEH361)
BC18018	dpy-5(e907)/dpy-5(e907);sEx18018(rCes Y75B8A.9::gfp+PcEH361)
BC18027	dpy-5(e907)/dpy-5(e907);sEx18027(rCes F18H3.1::gfp+PcEH361)
BC18032	dpy-5(e907)/dpy-5(e907);sEx18032(rCes T22A3.5::gfp+PcEH361)
BC18033	dpy-5(e907)/dpy-5(e907);sEx18033(rCes F33G12.5::gfp+PcEH361)
BC18035	dpy-5(e907)/dpy-5(e907);sEx18035(rCes R11H6.5::gfp+PcEH361)
BC18037	dpy-5(e907)/dpy-5(e907);sEx18037(rCes EEED8.9::gfp+PcEH361)
BC18055	dpy-5(e907)/dpy-5(e907);sEx18055(rCes F37A4.1::gfp+PcEH361)
BC18058	dpy-5(e907)/dpy-5(e907);sEx18058(rCes W09G3.7::gfp+PcEH361)
BC18059	dpy-5(e907)/dpy-5(e907);sEx18059(rCes K07A1.7::gfp+PcEH361)
BC18060	dpy5(e907)/dpy5(e907);sEx18060(rCesY46E12BL.3::gfp+PcEH361)
BC18062	dpy-5(e907)/dpy-5(e907);sEx18062(rCesF56D12.6a::gfp+PcEH361)
BC20271	dpy5(e907)/dpy5(e907);sEx20271(rCesY105E8A.14::gfp+PcEH361)
BC30135	dpy-5(e907)/dpy-5(e907);sEx30135(rCes C43G2.2::gfp+PcEH361)
